# Identification of small molecules that interfere with c-di-GMP signaling and induce dispersal of *Pseudomonas aeruginosa* biofilms

**DOI:** 10.1038/s41522-021-00225-4

**Published:** 2021-07-09

**Authors:** Jens Bo Andersen, Louise Dahl Hultqvist, Charlotte Uldahl Jansen, Tim Holm Jakobsen, Martin Nilsson, Morten Rybtke, Jesper Uhd, Blaine Gabriel Fritz, Roland Seifert, Jens Berthelsen, Thomas Eiland Nielsen, Katrine Qvortrup, Michael Givskov, Tim Tolker-Nielsen

**Affiliations:** 1 Costerton Biofilm Center. Department of Immunology and Microbiology, Faculty of Health and Medical Sciences, University of Copenhagen, Copenhagen, Denmark; 2 Department of Chemistry, Technical University of Denmark, Lyngby, Denmark; 3 Institute of Pharmacology and Research Core Unit Metabolomics, Hannover Medical School Carl-Neuberg-Straße 1, Hannover, Germany; 4 Singapore Centre for Environmental Life Sciences Engineering, Nanyang Technological University, Singapore, Singapore

**Keywords:** Biofilms, Antimicrobials

## Abstract

Microbial biofilms are involved in a number of infections that cannot be cured, as microbes in biofilms resist host immune defenses and antibiotic therapies. With no strict biofilm-antibiotic in the current pipelines, there is an unmet need for drug candidates that enable the current antibiotics to eradicate bacteria in biofilms. We used high-throughput screening to identify chemical compounds that reduce the intracellular c-di-GMP content in *Pseudomonas aeruginosa*. This led to the identification of a small molecule that efficiently depletes *P. aeruginosa* for c-di-GMP, inhibits biofilm formation, and disperses established biofilm. A combination of our lead compound with standard of care antibiotics showed improved eradication of an implant-associated infection established in mice. Genetic analyses provided evidence that the anti-biofilm compound stimulates the activity of the c-di-GMP phosphodiesterase BifA in *P. aeruginosa*. Our work constitutes a proof of concept for c-di-GMP phosphodiesterase-activating drugs administered in combination with antibiotics as a viable treatment strategy for otherwise recalcitrant infections.

## Introduction

Biofilms consist of densely packed bacteria concealed in shielding biopolymers and are often attached to surfaces. For billions of years, environmental bacteria have escaped extinction by forming biofilms. It has become clear that this capability also has a key role in the development of chronic and antibiotic-resistant infections^1^. In the biofilm mode, bacteria attain the highest levels of multiple resistances to our present assortment of antibiotics and antimicrobials, and exhibit an almost unlimited capability to evade the immune system and survive in the infected host^2^. The increasing population of elderly and hospitalized citizens has sparked a multitude of nosocomial infections that have become a major cause of death, disability, and social and economic upheaval for millions of people. Evidence is accumulating that such infections are caused by bacteria in the form of biofilms^3–5^. A major shortcoming of our current assortment of antibiotics is that they aim at bacteria present in an unshielded, planktonic, single-cell state. Thus, the previous antimicrobial discovery has used concentration-dependent inhibition of in vitro planktonic bacterial growth as the hallmark of the antimicrobial efficacy. Consequently, the majority of conventional antibiotics are not efficient for the treatment of biofilm infections.

Compelling evidence suggests that c-di-GMP signaling is a general and key bacterial process that controls the biofilm lifecycle^6,7^. A high internal level of c-di-GMP drives bacteria to form biofilms, whereas reduced c-di-GMP levels promote dispersal of biofilm bacteria, upon which they assume the planktonic mode of life. Diguanylate cyclase (DGC) enzymes catalyze the formation of c-di-GMP, whereas phosphodiesterase (PDE) enzymes catalyze the degradation of c-di-GMP. In response to a variety of environmental and chemical signals, a number of different DGC and PDE enzymes modulate the internal c-di-GMP content either by catalyzing the synthesis or the breakdown of c-di-GMP. We have previously provided evidence that induction of PDEs in *Pseudomonas putida* and *Pseudomonas aeruginosa* leads to biofilm dispersal^8–11^. In the case of *P. aeruginosa*, this was demonstrated in vitro as well as in a murine biofilm infection model^8,11^. Owing to the highly conserved nature of c-di-GMP signaling systems in Gram-negative bacteria and the strong evidence for their role in regulating biofilm formation, c-di-GMP signaling systems are promising targets for the development of drugs for the treatment of biofilm-associated infections in combination with antibiotics capable of killing planktonic bacteria^12–14^. A reduction in the c-di-GMP content in bacteria can be achieved by inhibiting precursors^15^, targeting the messenger^16^, inhibiting DGCs, or activating PDEs^17^. Several studies have focused on identifying compounds that inhibit DGCs. These include the development of c-di-GMP analogs that inhibit DGC activity^18–20^, identification of DGC inhibitors through in silico^21,22^ or in vitro screening of compound libraries^23–25^, as well as in vivo screening of compound libraries with bacterial assays^26–28^. Although stimulation of PDE activity may be more efficient at reducing the c-di-GMP content in bacteria than inhibition of DGC activity, studies that attempt to identify compounds that stimulate PDEs are rare. One notable example is the identification of NO as a compound capable of dispersing *P. aeruginosa* biofilms through activation of PDE activity^29–31^.

Here, we applied a high-throughput screening approach and tested 50,000 chemical compounds for their ability to reduce the content of c-di-GMP in *P. aeruginosa*. This resulted in the identification of a small molecule that dependent on the activity of a specific PDE induces dispersal of *P. aeruginosa* biofilms.

## Results

### Identification of a chemical compound that reduces the intracellular c-di-GMP content in *P. aeruginosa*

**Fig. 1:**
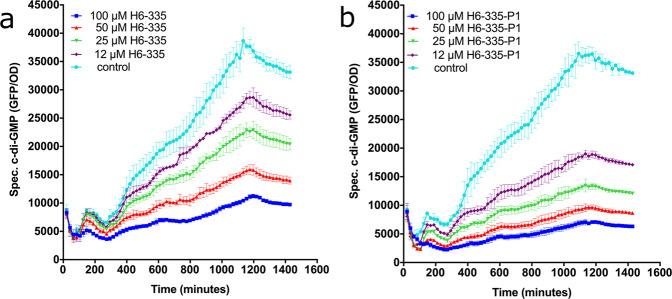
H6-335 and H6-335-P1 reduce the c-di-GMP content in *P. aeruginosa*. Effects of H6-335 (**a**) and H6-335-P1 (**b**) on the fluorescence output of the c-di-GMP monitor strain *P. aeruginosa* Δ*wspF*Δ*pel*Δ*psl/*pCdrA-gf*p*. Bacteria were grown in the wells of microtiter plates in the presence of various concentrations of H6-335 or H6-335-P1. GFP fluorescence was measured every 20 min for 24 h. Specific c-di-GMP levels (GFP/OD_600_) are plotted as a function of time and H6-335 or H6-335-P1 concentration. Mean and standard deviation (bars) of three biological replicates (*n* = 3) are shown.

We embarked on a high-throughput approach to screen a library of 50,000 synthetic compounds for the identification of molecules capable of significantly reducing the c-di-GMP content in *P. aeruginosa*. The screen was based on a *P. aeruginosa* Δ*wspF*Δ*pel*Δ*psl*/pCdrA-gfp strain, containing a plasmid-borne *cdrA-gfp* fusion that makes it capable of gauging the internal c-di-GMP content since expression from the *cdrA* promoter is positively regulated by c-di-GMP^32^. The Δ*wspF* mutation results in overproduction of c-di-GMP, which warrants a robust fluorescent output from the *cdrA-gfp* reporter fusion. The Δ*pel* and Δ*psl* gene deletions render the bacteria deficient in Pel and Psl exopolysaccharide synthesis, ensuring that the growing bacteria are in a single-cell planktonic mode, which enables reliable measurements of fluorescence and optical density. The screen resulted in the identification of a compound, designated H6-335, that significantly reduces the c-di-GMP content of the bacteria (Fig. 1a), without inhibiting the growth of the screening strain (Supplementary Fig. 1) nor the *P. aeruginosa* wild type (data not shown). At a concentration of 100 μM, H6-335 reduced the fluorescence output of the c-di-GMP monitor cultures by ~70% compared with the control that contained a medium supplemented with 1% dimethyl sulfoxide (DMSO) (Fig. 1a). DMSO was used as the non-treatment control in all experiments reported here, as it was used to dissolve H6-335 and its molecular derivatives.

### Compound validation and improvement of activity via SAR analysis

**Fig. 2:**
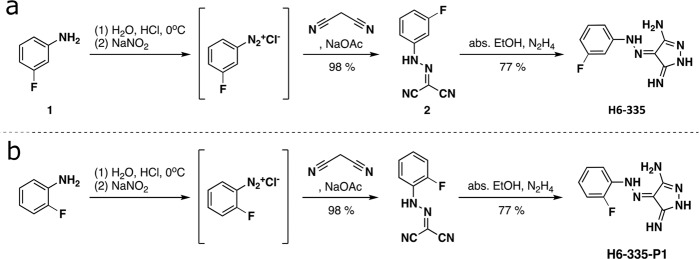
Chemical synthesis of H6-335 and H6-335-P1. Synthesis of (Z)-4-(2-(3-fluorophenyl)hydrazineylidene)-5-imino-4,5-dihydro-1H-pyrazol-3-amine (H6-335) (**a**) and 4-[(2-fluorophenyl) hydrazinylidene]pyrazole-3,5-diamine (H6-335-P1) (**b**) in a two-step procedure involving diazotation of the arylamines and condensation with malonodinitrile, followed by cyclocondensation with hydrazine.

**Fig. 3:**
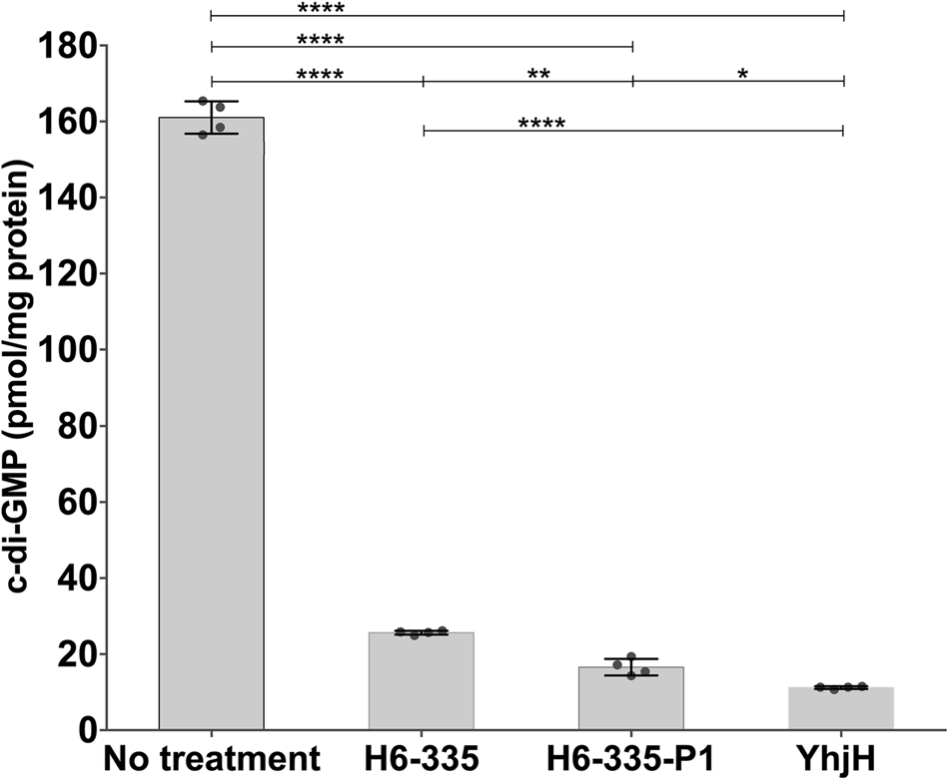
Effect of H6-335 and H6-335-P1 on the c-di-GMP level of *P. aeruginosa* Δ*wspF*Δ*pel*Δ*psl* determined by HPLC coupled MS-MS. Bacteria were grown as 25 ml cultures in 250 ml Erlenmeyer flasks in the presence of 100 μM H6-335, 100 μM H6-335-P1, or 0.05% DMSO as control. Following 8 h of growth (early stationary phase), samples for c-di-GMP and protein quantification were collected from each of the three cultures. *P. aeruginosa ∆wspF∆pel∆psl*/pYhjH^G^ containing plasmid pYhjH^G^-encoded YhjH phosphodiesterase was included as an additional control. Mean and standard deviation (bars) of c-di-GMP contents normalized to protein contents are shown for four biological replicates (*n* = 4). Significance levels are based on one-way ANOVA analysis with Sidak’s multiple comparisons test (*p < 0.05, **p < 0.01, ****p < 0.0001).

The identity of compound H6-335 was confirmed by chemical synthesis, starting from commercially available 3-fluoroaniline which was converted into the corresponding *N*-arylhydrazone, and subsequently cyclized with hydrazine to give the corresponding hydrazonodiaminopyrazole H6-335 (Fig. 2). Direct measurements of the intracellular pool of c-di-GMP in *P. aeruginosa* Δ*wspF*Δ*pel*Δ*psl* were performed by means of high performance liquid chromatography (HPLC) coupled tandem-MS analysis, which validated the above biological measurements of c-di-GMP contents. In line with the output measured by the live screen, 100 μM of H6-335 reduced the total intracellular c-di-GMP content of the *P. aeruginosa* Δ*wspF*Δ*pel*Δ*psl* strain by 84% compared with the DMSO-control (Fig. 3).

During substantial medicinal chemistry efforts, iterative rounds of structure-activity relationship (SAR) studies were performed to improve the potency and specificity of H6-335. A total of 65 H6-335 analogs, including compounds with a variation of the substitution pattern on the benzene ring in combination with various heterocycles, were synthesized and tested. As judged from the readout of the c-di-GMP gauging monitor strain, the compound H6-335-P1 (Fig. 2) was found to be the most active with respect to reduction of the c-di-GMP level in *P. aeruginosa* (Fig. 1b). The potency of the H6-335-P1 compound was confirmed with HPLC coupled tandem-MS analysis, which showed that 100 μM H6-335-P1 reduced the total cellular c-di-GMP content of the *P. aeruginosa* Δ*wspF*Δ*pel*Δ*psl* strain by 90% compared with the DMSO-control (Fig. 3). In accordance with the findings described above, the synthesized H6-335 and H6-335-P1 neither affected the growth rate nor the yield of the cultures (data not shown). A full account of compound design, chemical synthesis, and biological testing for all 65 H6-335 analogs will be published elsewhere.

In addition to determining c-di-GMP levels in *P. aeruginosa* Δ*wspF*Δ*pel*Δ*psl* exposed to H6-335 and H6-335-P1, we also determined c-di-GMP levels in the strain *P. aeruginosa* Δ*wspF*Δ*pel*Δ*psl*/pYhjH^G^, which expresses a plasmid-encoded YhjH PDE, originating from *Escherichia coli*. YhjH (also termed PdeH) is considered a strong PDE^33^, and we have previously demonstrated that induction of *yhjH* results in dispersal of *P. aeruginosa*/pYhjH biofilms^8^. The *P. aeruginosa* Δ*wspF*Δ*pel*Δ*psl*/pYhjH^G^ strain contained a level of c-di-GMP similar to the c-di-GMP level in the *P. aeruginosa* Δ*wspF*Δ*pel*Δ*psl* strain subjected to 100 μM H6-335-P1 (Fig. 3).

### H6-335-P1 inhibits *P. aeruginosa* biofilm formation

**Fig. 4:**
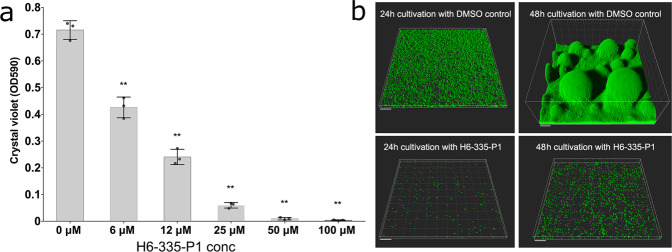
H6-335-P1-mediated inhibition of *P. aeruginosa* biofilm formation. **a** *P. aeruginosa* was cultivated for 10 h in the wells of microtiter plates in the presence of various concentrations of H6-335-P1. Subsequently, the amounts of biofilm in the wells were quantified using a crystal violet assay. Mean and standard deviation (bars) of three biological replicates (*n* = 3) are shown. One-way ANOVA analysis with Sidak’s multiple comparisons test was used to calculate significance values (**p < 0.01). **b** Gfp-tagged *P. aeruginosa* was cultivated in flow-cells perfused with growth medium with or without 25 μM H6-336-P1. CLSM micrographs of the adherent bacteria were acquired after 24 and 48 h of cultivation. Simulated 3D fluorescence projections were generated from the CLSM image stacks using IMARIS software. The size bars correspond to 20 μm.

Since the H6-335-P1 compound was capable of reducing the c-di-GMP content in *P. aeruginosa*, we expected that it would inhibit biofilm formation. We first investigated the effect of H6-335-P1 on *P. aeruginosa* biofilm formation in microtiter trays. The *P. aeruginosa* wild-type strain was grown for 10 h in microtiter trays in the presence of various concentrations of H6-335-P1, and the amount of biofilm in the wells was subsequently quantified by the use of crystal violet staining assay. As shown in Fig. 4a, H6-335-P1 inhibited biofilm formation in a concentration-dependent manner. The presence of 25 μM H6-335-P1 resulted in ~90% reduction in biofilm formation (Fig. 4a).

Next, we investigated the effect of H6-335-P1 on biofilm formation in flow-cells. In order to image the biofilms with confocal laser scanning microscopy (CLSM), we used a *P. aeruginosa* wild-type strain carrying a miniTn7-*gfp* insertion. Biofilms were grown in the presence of 25 μM H6-335-P1 or DMSO-control for 24 or 48 h and were then visualized by CLSM. The H6-335-P1 compound strongly inhibited biofilm formation in the flow-cells (Fig. 4b and Supplementary Fig. 2). Although the bacteria without H6-335-P1 formed a thick biofilm in the flow-cells during the 48 h of growth, the bacteria propagated in the presence of H6-335-P1 failed to form a biofilm and only a few single bacteria were found attached to the substratum.

### H6-335-P1 induces dispersal of *P. aeruginosa* biofilms

**Fig. 5:**
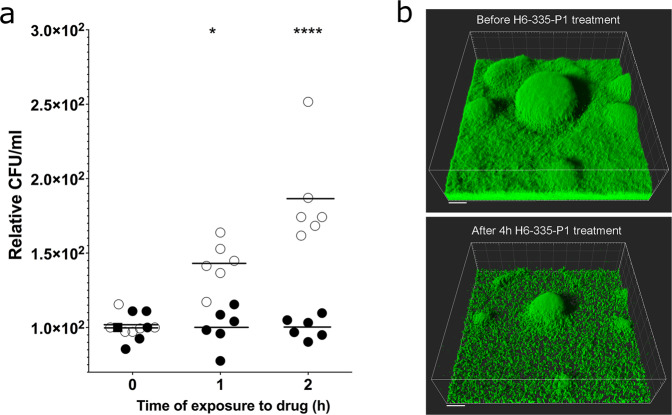
H6-335-P1-mediated dispersal of *P. aeruginosa* biofilms. **a** *P. aeruginosa* biofilms were grown in the wells of microtiter trays as 12 biological replicates (*n* = 12). After 18 h of cultivation 25 μM H6-335-P1 (*n* = 6, open circles) or 1% DMSO-control (*n* = 6, filled circles) was added. Subsequently, at the time-points indicated, samples were withdrawn and plated for CFU determinations. Average CFU’s per ml for the DMSO controls at each time point were set to 100. Horizontal bars represent mean values. Two-way ANOVA analysis with Sidak’s multiple comparisons test was used to calculate significance values (*p < 0.05, ****p < 0.0001). **b** A biofilm of Gfp-tagged *P. aeruginosa* was cultivated in a flow-cell irrigated with growth medium supplemented with 0.025% DMSO. After 48 h of cultivation, the flow-through medium was shifted so that it contained 0.025% DMSO and 25 μM H6-335-P1. CLSM micrographs were acquired before and 4 h after the introduction of H6-336-P1. Simulated 3D fluorescence projections were generated from the CLSM image stacks using IMARIS software. The size bars correspond to 20 μm.

We then investigated the effect of addition of H6-335-P1 to established biofilms in microtiter tray wells. Wild-type bacteria were grown in microtiter trays for 18 h, upon which 25 μM H6-335-P1 or DMSO-control was added. Liquid samples from the wells were collected at 0, 1, and 2 h after addition of H6-335-P1 or DMSO, and colony-forming units (CFU/ml) were quantified for each sample. We found a significant increase in CFU/ml in the liquid of the wells that had received H6-335-P1 compared with the wells that had received the DMSO-control (Fig. 5a). Thus, H6-335-P1 is capable of inducing dispersal of established biofilms in microtiter trays.

We also examined the effect of adding H6-335-P1 to *P. aeruginosa* biofilms grown in flow-cells. We grew the *gfp*-tagged *P. aeruginosa* wild-type strain for 48 h in flow-chambers irrigated with medium containing DMSO-control, and then shifted to medium with 25 μM H6-335-P1. The biofilms were imaged by CLSM immediately before the medium shift, and after 4 h of exposure to H6-335-P1. As shown in Fig. 5b and Supplementary Fig. 3, the addition of H6-335-P1-induced significant dispersal of the flow-cell grown biofilm.

### H6-335-P1 improves subsequent antibiotic kill of biofilms and dispersed bacteria

**Fig. 6:**
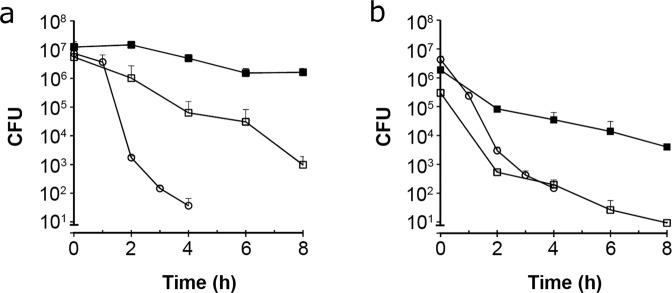
Antibiotic time-kill assay of *P. aeruginosa* biofilms and dispersed cells. Biofilms were grown on pegs for 24 h, and were subsequently treated with 100 μM H6-335-P1 or DMSO-control for 2 h. Biofilms treated with H6-335-P1 (□) and DMSO-control (■), as well as dispersed cells (〇) were then challenged with 30 µg/ml tobramycin (**a**) or 0.5 µg/ml ciprofloxacin (**b**). The biofilms were disrupted at 0, 2, 4, 6, and 8 h, and planktonic cells were withdrawn at 0, 1, 2, 3, and 4 h, and the bacteria were plated on agar plates for CFU determination. Mean values and standard deviation (bars) of three biological replicates (*n* = 3) are shown.

We subsequently investigated the hypothesis that an efficient biofilm dispersing compound would promote the killing efficacy of conventional antibiotics. To this end, *P. aeruginosa* biofilms were cultivated for 24 h on polystyrene pegs protruding down from plastic lids into the wells of microtiter plates. The biofilm-coated pegs were then submerged for 2 h in medium supplemented with either 100 μM H6-335-P1 or DMSO-control. Thereafter, the biofilms on the pegs and the dispersed planktonic bacteria in the microtiter tray wells were treated separately with tobramycin or ciprofloxacin. The efficacy of the antibiotic treatment on the planktonic cells was assessed by withdrawing samples for CFU determination after 0, 1, 2, 3, and 4 h. The effect of the antibiotic treatment on the biofilm on the pegs was assessed by dispersing the biofilms via sonication after 0, 2, 4, 6, and 8 h, followed by CFU determination. The experiment demonstrated that dispersed planktonic cells were rapidly killed in a time-dependent manner (Fig. 6). On the contrary, the antibiotic treatment only showed a marginal killing effect on biofilms that had not been treated with H6-335-P1, whereas the antibiotics were more efficient on the bacteria that remained attached to the pegs after H6-335-P1 treatment (Fig. 6). The latter result indicates that improved access to the biofilm bacteria that were not liberated by H6-335-P1 exposure promotes antibiotic-mediated killing. In conclusion, our experiments indicate that our biofilm dispersing agent increases the efficacy of conventional antibiotics.

### H6-335-P1 treatment significantly reduces *P. aeruginosa* biofilm infections in mice

**Fig. 7:**
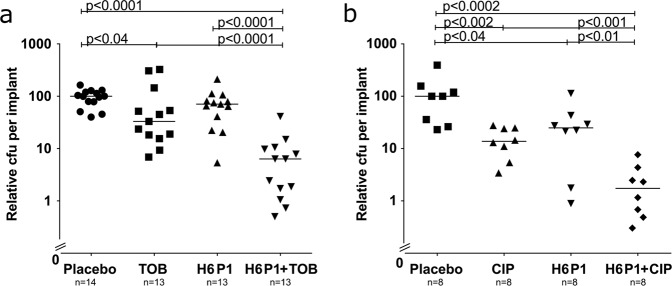
Treatment of biofilm infections with H6-335-P1 and antibiotics. Silicone implants were incubated with *P. aeruginosa* cultures for 20 h for bacterial adhesion. At time zero, mice had implants inserted in the peritoneal cavity. At 24 h and 26 h post insertion of the implants (PI), the mice were treated with either H6-335-P1 (H6 P1) (25 μM corresponding to 6 μg compound per gram of bodyweight) or vehicle (as placebo), and at 26 h PI the mice received 30 μg tobramycin (TOB) per gram bodyweight or 0.9% NaCl (**a**), or alternatively at 24 h PI the mice received 10 μg ciprofloxacin (CIP) per gram bodyweight or 0.9% NaCl (**b**). At 28 h PI, the mice were euthanized and the CFU per implant was determined. The median CFU per implant for the placebo group was set to 100. The median CFU per implant for the placebo groups were 8.5 × 10^6^ (**a**) and 1.5 × 10^6^ (**b**). Horizontal lines indicate median CFU for each group. Each symbol represents a mouse. Significance levels are based on Mann–Whitney *U* test (analysis of non-parametric data).

Subsequently, we determined the anti-biofilm efficacy of H6-335-P1 in mice that were installed with bacteria-coated implants. *P. aeruginosa* biofilms were allowed to form for 24 h on implants located in the mouse peritoneal cavity, after which the mice were given either placebo or H6-335-P1 as intraperitoneal injections. Dosages corresponding to 1–6 μg compound per gram of bodyweight were administered to the mice. After the treatments, the implants were removed and the bacteria remaining on the implants were enumerated as CFU. Similar to the outcome of the in vitro investigations described above, 4 hours of exposure to H6-335-P1 reduced the number of bacteria on the implants by ~90% (Supplementary Fig. 4). Next, we conducted a series of experiments with combinatorial treatments with H6-335-P1 and antibiotics. Biofilms developed on the implants during 24 h of insertion, after which the mice were given either placebo or H6-335-P1 at 24 h and 26 h post insertion (PI). In addition, either tobramycin (26 h PI) or ciprofloxacin (24 h PI) was administered to the mice, also as intraperitoneal injections. Subsequently, CFU enumeration of removed implants demonstrated that combinatorial treatment with H6-335-P1 and antibiotic displayed enhanced antimicrobial effects (Fig. 7). Our results indicate that the significant reduction in the biofilm mass resulting from the H6-335-P1 treatments offers improved access for the subsequently administered antibiotics, which results in an improved kill of the bacteria. We note that the tobramycin was administered 2 hours after H6-335-P1, whereas ciprofloxacin was administered at the same time as H6-335-P1, and in both cases the combination of anti-biofilm compound and antibiotic were efficient.

### The c-di-GMP PDE BifA is central for H6-335-P1 activity

Since the *P. aeruginosa* strain used for our screen is a *wspF* mutant with increased activity of the WspR DGC, we initially expected that WspR was the target of H6-335-P1. However, we found that H6-335-P1 could efficiently inhibit biofilm formation of a *P. aeruginosa wspR* mutant, indicating that the reduction of the c-di-GMP content is not due to inhibition of WspR (data not shown). We employed mutational analysis with the aim of identifying a putative target of H6-335-P1. The compound Congo Red (CR) binds to exopolymers whose production is positively regulated by c-di-GMP in *P. aeruginosa*^34^. Thus, we exploited that a *P. aeruginosa wspF* mutant forms wrinkled dark red colonies when cultivated on agar plates with CR, but forms smooth white colonies when cultivated on CR plates supplemented with 100 μM H6-335-P1 (Supplementary Fig. 5). We constructed a mariner transposon mutant library in the *P. aeruginosa wspF* background and spread the resulting 32,000 transposon mutants onto CR plates supplemented with H6-335-P1. Two days later, visual inspection of the agar plates revealed that the large majority of the transposon mutants gave rise to smooth white colonies as expected, whereas 260 mutants displayed a wrinkled red colony phenotype despite the presence of H6-335-P1. Biofilm inhibition assays performed on all 260 mutants showed that only one of the mutants no longer responded to the presence of H6-335-P1 and formed biofilms similar to the control that was not treated with H6-335-P1 (data not shown). We sequenced and determined the transposon insertion point in the mutant that did not respond to H6-335-P1 as well as in 99 of the other mutants that formed red colonies on CR plates with H6-335-P1. In the mutant that did not respond to H6-335-P1 the transposon resided in the coding sequence of the *bifA* gene. The *bifA* gene encodes a well-characterized PDE, which has been demonstrated to be a key player in c-di-GMP signaling and biofilm formation in *P. aeruginosa*^35^. In the 99 other mutants that formed red colonies on agar plates with CR and H6-335-P1, the transposon insertions were found to reside in genes mainly involved in lipopolysaccharide and polysaccharide synthesis or their regulatory functions (data not shown). Apparently, all these mutations result in the formation of red colonies on agar plates with CR and H6-335-P1, but only the *bifA* mutation resulted in an inability to respond to H6-335-P1 in biofilm formation assays.

Subsequently, we constructed a clean *bifA* knockout mutant in the *P. aeruginosa wspF* background, and could demonstrate that the constructed Δ*bifA*Δ*wspF* mutant no longer responded to the presence of H6-335-P1, but formed biofilms similar to the control that was not treated with H6-335-P1 (Supplementary Fig. 6A). We also knocked out *bifA* in the *P. aeruginosa* Δ*wspF*Δ*pel*Δ*psl*/pCdrA-gfp c-di-GMP monitor strain, and found that the fluorescent readout from the Δ*bifA*Δ*wspF*Δ*pel*Δ*psl*/pCdrA-gfp strain was completely unaffected by the presence of H6-335-P1, whereas 100 μM H6-335-P1 reduced the GFP readout from the *P. aeruginosa* Δ*wspF*Δ*pel*Δ*psl*/pCdrA-gfp monitor bacteria significantly (Supplementary Fig. 6B).

**Fig. 8:**
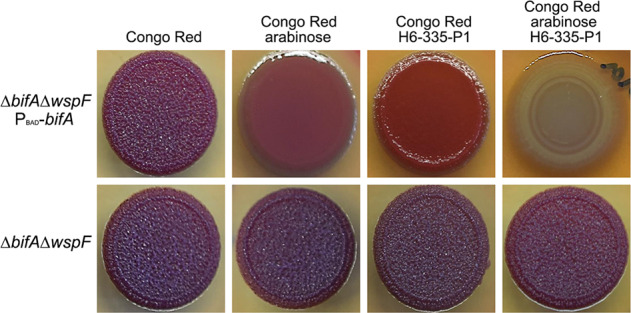
The presence of H6-335-P1 results in activation of the BifA protein. Macrocolonies were grown from 5 μl overnight culture of *P. aeruginosa* Δ*bifA*Δ*wspF* P_BAD_-*bifA* or *P. aeruginosa* Δ*bifA*Δ*wspF* spotted on agar plates supplemented with, respectively, Congo Red, Congo Red and arabinose, Congo Red and H6-335-P1, or Congo Red, arabinose and H6-335-P1. Photographs of the macrocolonies were acquired after 48 h incubation at 30°C.

Next, we complemented the *P. aeruginosa* Δ*bifA*Δ*wspF* mutant by insertion in the ϕCTX site of a *bifA* gene fused to the arabinose-inducible P_BAD_ promoter. As shown in Fig. 8, this strain formed wrinkled dark red colonies on agar plates with CR, whereas it formed smooth and lighter red colonies on plates supplemented with both CR and arabinose. The complemented strain also formed smooth red colonies on agar plates containing CR and H6-335-P1 (Fig. 8), which is probably due to leakiness of the P_BAD_ promoter. Accordingly, the non-complemented *P. aeruginosa* Δ*bifA*Δ*wspF* strain formed wrinkled dark red colonies on agar plates with CR and H6-335-P1 (Fig. 8). Importantly, however, the *P. aeruginosa* Δ*bifA*Δ*wspF* P_BAD_-*bifA* strain formed white colonies on agar plates containing both CR, arabinose and H6-335-P1 (Fig. 8). The P_BAD_-*bifA* fusion does not contain the native *bifA* promoter, and thus the level of BifA protein in the bacteria is determined by arabinose-induced expression from the P_BAD_ promoter. Therefore, we anticipate that the bacteria that were exposed to both arabinose and H6-335-P1 had a similar level of BifA protein as the bacteria that were exposed to arabinose only. Yet, the bacteria formed red colonies on the agar plates with CR and arabinose, but white colonies on the agar plates with CR, arabinose, and H6-335-P1 (Fig. 8). These results indicate that the presence of H6-335-P1 results in activation of the BifA protein.

**Fig. 9:**
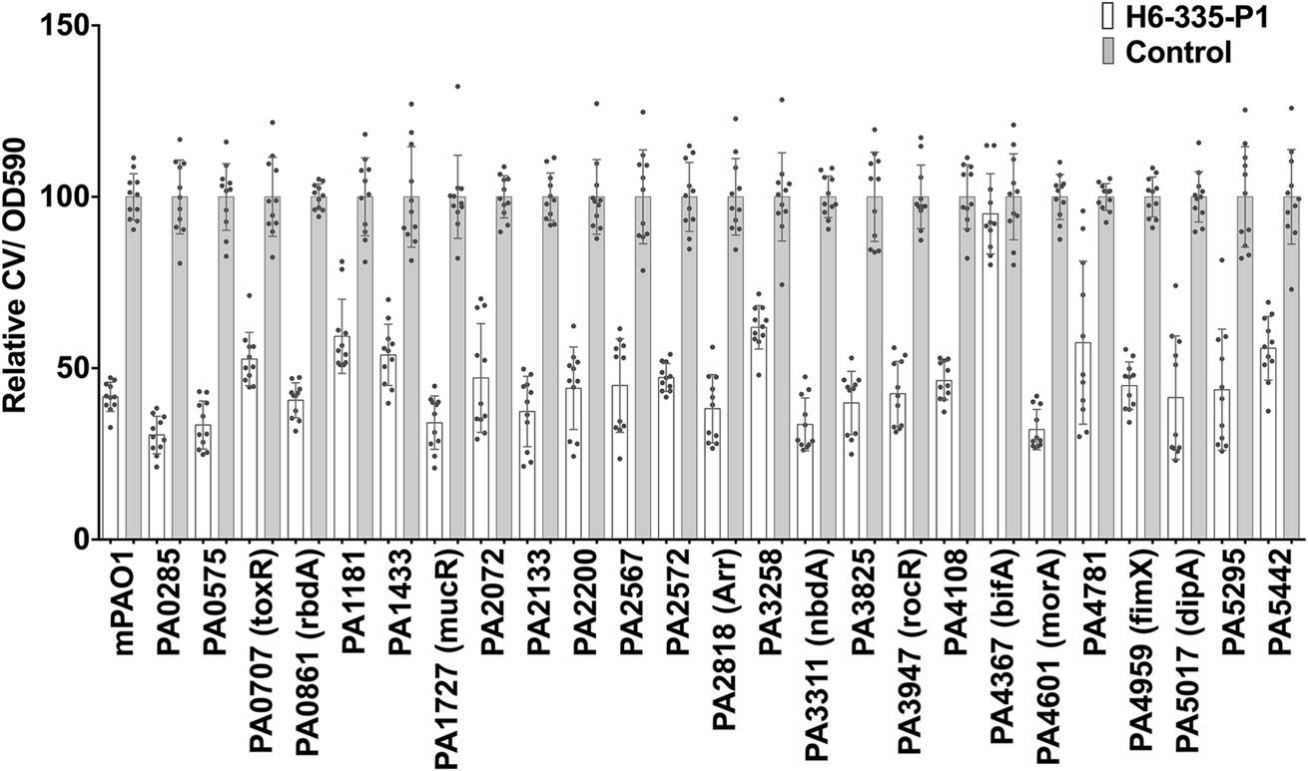
A functional BifA is required for H6-335-P1-induced dispersal of *P. aeruginosa* biofilm. Biofilms of the *P. aeruginosa* wild-type and PDE mutants were grown in the wells of microtiter trays for 18 h, at which time point 100 μM H6-335-P1 or DMSO-control was added to the wells. After 2 hours of further incubation, the culture supernatants were discarded and the amount of biofilm present in the wells was quantified by crystal violet staining. The graph shows relative values, where the average crystal violet value of each DMSO-control was set to 100. Mean value and standard deviation (bars) of 11 biological replicates (*n* = 11) are shown. One-way ANOVA analysis with Sidak’s multiple comparisons test was used to calculate significance values between control and H6-335-P1-treated. *P* < 0.0001 was found for all comparisons, except PA4367 (*bifA*) for which H6-335-P1-treated was not significantly different from the control (*p* > 0.05).

The finding that biofilm formation and c-di-GMP level of the *P. aeruginosa bifA* mutants was completely unaffected by the presence of H6-335-P1, indicates that BifA is the primary c-di-GMP-metabolizing enzyme that is affected by H6-335-P1 exposure. To corroborate this, we investigated whether other PDEs might be involved in reducing the intracellular c-di-GMP content in response to H6-335-P1 exposure. We obtained mutants of all putative *P. aeruginosa* PDEs from the Washington *P. aeruginosa* PAO1 transposon mutant library^36^ and investigated the ability of H6-335-P1 to disperse biofilms formed by each of these mutants in microtiter trays. As shown in Fig. 9, the *bifA* mutant biofilm did not respond to H6-335-P1, whereas the biofilms formed by the *P. aeruginosa* wild type and the remaining PDE mutants dispersed in response to H6-335-P1 exposure. Several of the PDE mutants formed more biofilm than the *bifA* mutant (data not shown), indicating that BifA is not the most potent PDE in the wild type, which could have been an alternative explanation for the BifA dependency of H6-335-P1-mediated reduction of the c-di-GMP content in *P. aeruginosa*.

The experiments described above show that the c-di-GMP-reducing and anti-biofilm activity of H6-335-P1 occurs through the BifA PDE in *P. aeruginosa*. Although our mutant screen indicated that BifA is the direct target of H6-335-P1, we cannot exclude that H6-335-P1 could stimulate BifA activity indirectly, e.g., by activation of a protein that interacts with BifA. To discriminate between these two possibilities, we attempted to perform in vitro PDE activity assays with purified BifA. However, the BifA we could purify by His-tagging and expression in *E. coli* did not show PDE activity in vitro (data not shown) and, consequently, we could not investigate directly if the presence of H6-335-P1 stimulated the activity of BifA. Alternatively, we investigated whether H6-335-P1 could stimulate BifA expressed ectopically in *E. coli*. There are no *bifA* orthologs in *E. coli*, and therefore it is highly unlikely that *E. coli* has evolved proteins that can interact specifically with BifA. Accordingly, H6-335-P1-mediated activation of ectopically expressed BifA in *E. coli* would strongly indicate that BifA is the direct target of H6-335-P1. We transformed *E. coli* AR3110 with, respectively, the pJN105 vector and plasmid pJN105::bifA^11^, enabling ectopic expression of BifA by induction with arabinose. The *E. coli* AR3110 strain has intact cellulose biosynthesis genes and forms red colonies on CR agar plates, as the cellulose binds CR^37^. Because cellulose production in *E. coli* is c-di-GMP regulated^38^, a reduction of the c-di-GMP level in *E. coli* will reduce the red color of the colonies on CR agar plates. We found that the presence of H6-335-P1 reduced the red color of *E. coli* AR3110/pJN105::bifA colonies on agar plates with CR and arabinose (Supplementary Fig. 7A). We noted that the presence of arabinose also appeared to reduce cellulose production in *E. coli* (Supplementary Fig. 7A). However, the presence of both H6-335-P1 and arabinose reduced the red color more than the presence of arabinose alone (Supplementary Fig. 7A). In our attempts to purify BifA, we cloned a truncated gene encoding the cytoplasmic part of BifA into the pQE30 vector resulting in plasmid pQE30::bifA180, which enables isopropyl β-d-1-thiogalactopyranoside-induced expression of BifA180. The results shown in Supplementary Figure 7B indicate that H6-335-P1 also reduced cellulose production by *E. coli* AR3110/pQE30::bifA180 expressing the cytoplasmic part of BifA. Together these results indicate that H6-335-P1 can activate ectopically expressed BifA in *E. coli*. Together, the experiments described above suggest that BifA is the direct target of H6-335-P1.

### RNA-seq analysis of the effect of H6-335-P1 on the transcriptome of *P. aeruginosa*

**Fig. 10:**
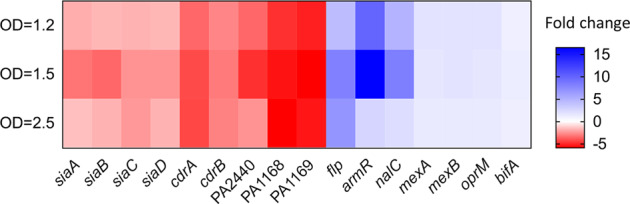
Heat-map showing the expression level of selected genes determined by RNA-seq analysis of H6-335-P1-treated *P. aeruginosa ΔwspFΔpelΔpsl* compared with untreated control. The results are based on three biological experiments (*n* = 3). OD: Cell densities measured as OD_600_ values.

RNA-sequencing (RNA-seq) was used to analyze gene expression profiles of *P. aeruginosa* cultures that were treated with H6-335-P1. The analysis was done on a *P. aeruginosa* Δ*wspF*Δ*pel*Δ*psl* strain and on a wild-type strain to determine the effects of H6-335-P1 on a high c-di-GMP level strain as compared with the wild type strain with lower levels of c-di-GMP. Liquid cultures were grown in shaken culture flasks and 50 μM H6-335-P1 or DMSO-control was added to the cultures at an optical density of 0.5, upon which samples for RNA-seq were retrieved after 1.5, 2.5, and 3.5 h of further incubation, corresponding to the mid-log, early stationary, and stationary growth phase. The transcriptomic analysis revealed that H6-335-P1 affected the expression of a number of genes in the *P. aeruginosa* Δ*wspF*Δ*pel*Δ*psl* strain, whereas the wild-type strain was less affected (the RNA-Seq data have been deposited in the NCBI Sequence Read Archive database with accession code PRJNA673995). This indicates that the effect of H6-335-P1 is more pronounced for bacteria with high c-di-GMP levels such as wild-type *P. aeruginosa* in the biofilm mode of growth, whereas the effect is less pronounced on bacteria with low c-di-GMP levels such as wild type *P. aeruginosa* bacteria in the planktonic mode of growth. Except for *siaD*, H6-335-P1 exposure did not affect the transcription of any of the multiple PDE and DGC encoding genes, including the *bifA* gene. Expression of the *siaABCD* operon was decreased in the *P. aeruginosa* Δ*wspF*Δ*pel*Δ*psl* strain in response to H6-335-P1 exposure (Fig. 10). The *siaD* gene encodes a DGC, but we found that H6-335-P1 could reduce the c-di-GMP level in a Δ*siaD*Δ*wspF*Δ*pel*Δ*psl* strain as efficiently as in the Δ*wspF*Δ*pel*Δ*psl* strain (Supplementary Fig. 8), indicating that downregulation of *siaD* does not contribute significantly to the reduction of the c-di-GMP content caused by H6-335-P1. As expected, H6-335-P1 exposure significantly reduced expression of *cdrA*, *cdrB,* and *PA2440* in the *P. aeruginosa* Δ*wspF*Δ*pel*Δ*psl* strain (Fig. 10), which was validated by subsequent qPCR analysis (data not shown). Besides, a number of other genes known to be responsive to a change in the c-di-GMP level^39,40^ were found to respond as expected. It was not possible to monitor changes in gene expression of *pel* and *psl* because of the *pel* and *psl* deletions in the Δ*wspF*Δ*pel*Δ*psl* strain (which ensured that the bacteria could be grown in planktonic culture despite having a high level of c-di-GMP). The expression of the genes *nalC* and *armR* was increased in the *P. aeruginosa* Δ*wspF*Δ*pel*Δ*psl* strain in response to H6-335-P1 exposure (Fig. 10). Both genes have been shown to be involved in the regulation of the multidrug efflux transporter MexAB-OprM. However, the transcription of the efflux transporter was not affected by treatment with H6-335-P1 (Fig. 10), and no change in resistance to ciprofloxacin was found in cultures treated with H6-335-P1 (data not shown). The transcription of the gene *flp*, encoding a subclass of type IVb pili known as Flp pili, was increased in response to H6-335-P1 exposure (Fig. 10). The *flp* gene is a part of the *tad* locus consisting of 13 genes, for which evidence has been provided that they are involved in adhesion to abiotic surfaces and eukaryotic cells^41^. The transcription of eight of the genes in the locus was increased in response to H6-335-P1 exposure. However, our studies of the effect of H6-335-P1 on biofilm formation of *P. aeruginosa* in microtiter trays and flow-cells indicate that attachment to abiotic surfaces is decreased in response to H6-335-P1 treatment. Transcription of the operon consisting of PA1168 and PA1169 was significantly decreased in cultures exposed to H6-335-P1. PA1169 encodes the lipoxygenase LoxA^42^, which has been shown to be induced during biofilm growth^43,44^. Furthermore, LoxA was found to be highly expressed in clinical *P. aeruginosa* isolates from cystic fibrosis lungs^43^, and to increase bacterial survival in lung tissue in a mouse model by modulating the host immune response^44^. Genes encoding other known virulence factors were unresponsive to H6-335-P1 exposure.

### H6-335-P1 is a narrow range anti-biofilm compound

A bioinformatics analysis showed that the *bifA* gene is conserved among the pseudomonads but is not widespread in other bacterial species. Accordingly, we found that H6-335-P1 did not induce dispersal of biofilms formed by the ESKAPE pathogens *Enterococcus facieum, Staphylococcus aureus*, *Klebsiella pneumoniae, Acinetobacter baumanii*, and *Enterobacter cloacae* (data not shown). In addition, H6-335-P1 did not induce dispersal of biofilms formed by other important opportunistic pathogens such as *Escherichia coli, Burkholderia cenocepacia,* and *Stenotrophomonas maltophilia* (data not shown). Taken together with the highly restricted structural freedom to maintain biological activity as indicated by the SAR analysis, the effects of H6-335-P1 are likely to be restricted to pseudomonads.

## Discussion

We employed a high-throughput screening approach to identify molecules that reduce the c-di-GMP content in *P. aeruginosa* and, thereby, inhibit biofilm formation and disperse already formed biofilm. The mode of action of the identified c-di-GMP-reducing compounds, H6-335 and H6-335-P1, depends on stimulation of the activity of the PDE BifA. In contrast, previous attempts to identify c-di-GMP-reducing compounds have focused on the identification of compounds that inhibit the activity of DGCs^15,22,26^. We found that our compounds efficiently reduced the c-di-GMP content in *P. aeruginosa* and displayed strong anti-biofilm activity both in vitro and in vivo. Thus, the work described here can be regarded as a proof of concept for PDE stimulation as a viable anti-biofilm strategy. Although the activity of the H6-335 compounds is limited to activation of BifA, it is conceivable that compounds activating a broader spectrum of PDEs can be identified.

A key structural motif of the *P. aeruginosa* anti-biofilm compounds identified in this study, H6-335 and H6-335-P1, is a substituted pyrazole. This heterocyclic core structure is present in a range of pharmaceutically relevant and biologically active compounds with anticancer^45–47^, anti-inflammatory^48^, anti-HIV^49^, anti-viral^50^, anti-diabetic^51^, anti-tubercular^52^, and anti-bacterial^53^ properties. For the subclass of hydrazonodiaminopyrazole-type compounds, Kojic et al. have demonstrated how 4-(4-fluoro-3-trifluoromethylphenyl)hydrazono 4H-pyrazole-3,5-diamine displays an anti-inflammatory effect through integrin-linked kinase inhibition^54,55^. Moreover, Kryštof et al. identified the compound 4-[(3,5-diamino-1H-pyrazol-4-yl)diazenyl]phenol as a selective inhibitor of cyclin-dependent kinase 9 (CDK9) in vitro^56,57^. CDK enzymes play essential roles in the regulation of cell division in humans and as such, the development of specific CDK inhibitors may result in novel anticancer agents. However, the anti-biofilm activity discovered for the hydrazonodiaminopyrazole compounds in the present study is novel.

Although direct biochemical proof is lacking, our experiments suggest that H6-335-P1 specifically activates the PDE BifA in *P. aeruginosa*. Activation of a PDE is remarkable from a broader pharmacological perspective. Almost all drugs acting on enzymes are inhibitors^58^. Non-specific PDE inhibitors such as theophylline, selective PDE3 inhibitors such as milrinone, and selective PDE5 inhibitors such as sildenafil are used clinically^58^. Enzyme activation has so far been realized pharmacologically relevant only in a few cases. Most notable are allosteric nitric oxide-dependent stimulators of soluble guanylyl cyclase (sGC) and allosteric nitric oxide-independent activators of sGC^59,60^. These drug classes are used for pulmonary arterial hypertension and studied for a number of other diseases. In contrast, allosteric activation of membranous adenylyl cyclases (mAC) by the diterpene forskolin has not yet reached significant clinical relevance because of the lack of AC isoform-specificity of forskolin analogs^61^. In the broad field of anti-pathogenic drugs, enzyme activators are very uncommon as well; the pharmacopoeia is dominated by enzyme inhibitors^58^. Our study highlights the importance of systematically searching not only for enzyme inhibitors but also for enzyme activators. This emphasizes that drug-screening assays should be designed in such a way that they can detect both enzyme inhibition and enzyme activation^62^.

Through the use of PDE genes engineered with inducible promoters, we have previously demonstrated that induction of PDEs inhibits *P. aeruginosa* biofilm formation and results in dispersal of already established *P. aeruginosa* biofilms^8,11^. On the contrary, Sauer and coworkers reported that induction of native PDEs does not lead to dispersal of *P. aeruginosa* biofilms^63–65^. In the present study, we provide evidence that H6-335-P1-mediated activation of BifA is sufficient to induce dispersal of *P. aeruginosa* biofilms, which is in accordance with our previous findings^11^. In our previous study, we used two different *P. aeruginosa* strains with inducible native PDE genes; one that harbored a *P*_*BAD*_*-bifA* fusion and one that harbored a *P*_*BAD*_*-PA2133* fusion. We found that induction of *P*_*BAD*_*-PA2133* more efficiently reduced the c-di-GMP level and prevented/dispersed *P. aeruginosa* biofilm than induction of *P*_*BAD*_*-bifA*^11^. The results obtained in the present study also indicate that induction of the *P*_*BAD*_*-bifA* fusion results in relatively modest PDE activity. Thus, induction of *P*_*BAD*_*-bifA* in a Δ*wspF*Δ*bifA* background was sufficient to mediate a change from a red wrinkly colony morphology to a red smooth colony morphology on CR agar plates, whereas an additional stimulation of BifA activity with H6-335-P1 was necessary to obtain white colonies on CR plates (Fig. 8). Although BifA seems to be a relatively weak PDE, the results presented here indicate that H6-335-P1-mediated activation of BifA results in a very efficient reduction of the c-di-GMP level in *P. aeruginosa*, and disperse *P. aeruginosa* biofilms.

According to the current model, sensing of biofilm dispersal cues by *P. aeruginosa* involves the chemotaxis-like methyl-accepting chemotaxis protein homolog BdlA, the PDEs DipA, RbdA, MucR, and NbdA, and the DGCs GcbA and NicD^65^. However, we found that H6-335-P1 was capable of dispersing *P. aeruginosa* biofilms formed by *dipA*, *rbdA*, *mucR,* and *nbdA* mutants (Fig. 9). Moreover, we have shown that H6-335-P1 can reduce the c-di-GMP content in a *bdlA* mutant as efficiently as in the wild type (Supplementary Fig. 8). These results suggest that H6-335-P1-induced biofilm dispersal occur independent of the currently accepted biofilm dispersal pathway, and support a model in which it occurs solely by stimulation of BifA.

Our transcriptomic analysis was performed on batch cultures with matching growth conditions and kinetics. In order to keep the *wspF* mutant bacteria in a truly planktonic state, the test strain was devoid of *psl* and *pel* genes. With these stringent precautions taken to mitigate pleotropic effects on gene expressions, exposure of *P. aeruginosa* to H6-335-P1 only showed modest effects on global gene expression profiles. In particular, no effect on virulence expression, including quorum sensing-regulated genes, was observed. This is in contrast to a previous study, which indicated that overexpression of the *E. coli* PDE YhjH in *P. aeruginosa* increased the expression of quorum sensing-regulated genes^66^. Whereas differences in growth conditions allegedly affects global gene expression profiles, evidence is emerging that c-di-GMP signaling-specificity, in addition to the c-di-GMP level, depends on protein–protein interactions^33,67^. Thus, overexpression of YhjH may lead to a different cellular output than activation of BifA.

The anti-biofilm compound H6-335-P1 is specific for *P. aeruginosa*, and might serve as a lead compound for the development of drugs that can be used in combination with antibiotics to treat a number of *P. aeruginosa* biofilm infections, including cystic fibrosis lung infections, chronic wound infections, urinary tract infections, and ventilator-associated pneumonia. The work presented here may also be regarded as proof of concept for PDE activation as an anti-biofilm strategy and may encourage a quest for other PDE activators, including broad-spectrum PDE activators.

## Methods

### Bacterial strains, plasmids, primers, and growth media

The *P. aeruginosa* and *Escherichia coli* strains used in this study are listed in Supplementary Table 1. The growth media employed for propagation of the strains were either ABTrace medium^68^ or lysogeny broth (LB). The growth medium used for CR plate assays was LB supplemented with 1% agar (Difco), 20 µg/ml coomassie blue, 40 µg/ml CR, 0.1% DMSO and either 0.2% (w/v) l-arabinose, 100 µM H6-335-P1, or 0.2% (w/v) l-arabinose together with 100 µM H6-335-P1. When necessary, growth media were supplemented with the following antibiotics: gentamicin, 10 µg/ml for *E. coli* and 60 µg/ml for *P. aeruginosa*. Tetracycline, 10 µg/ml for *E. coli* and 60 µg/ml for *P. aeruginosa*. Ampicillin 100 µg/ml for *E. coli*. Carbenicillin, 200 µg/ml for *P. aeruginosa*. Ciprofloxacin and tobramycin as indicated in the text for *P. aeruginosa*. Plasmids and primers used in this study are listed in Supplementary Tables 2 and 3, respectively.

### Chemical synthesis of H6-335 and H6-335-P1

The 4-arylazo-3,5-diamino-1H-pyrazole core structure of H6-335 and H6-335-P1 was assembled through a two-step synthetic procedure^56^ starting with diazotization of a suitable aniline to form the corresponding diazonium ion, which was then treated with malononitrile to give the *N*-phenylcarbonohydrazonoyl dicyanide intermediate. The second step was a reaction with hydrazine to give the corresponding 4-arylazo-3,5-diamino-1H-pyrazole analogs.

### Screening for compounds that reduce the c-di-GMP level in *P. aeruginosa*

A robot-assisted high-throughput procedure was used to screen 50,000 chemical compounds for their ability to reduce the c-di-GMP content in the *P. aeruginosa* Δ*wspF*Δ*pel*Δ*psl*/pCdrA-gfp c-di-GMP monitor strain^32^. In 96-well microtiter plates, overnight cultures of the monitor strain were diluted 100-fold into 100 µl ABTrace medium aliquots^68^ supplemented with 0.2% glucose, 0.5% casa amino acids, 60 µg/ml gentamicin, and 1 µM FeCl_3_. Using a liquid handling robot, the 50,000 test-compounds dissolved in DMSO were each dispensed into a well in 96-well microtiter plates (final concentration of 100 µM compound and 1% DMSO). Fifty µM sodium nitroprusside was used as positive control and 1% DMSO as negative control, both were included in all microtiter plates. The microtiter plates were incubated at 37°C on a rotary shaker (280 RPM for 18 h), after which 100 μl of sterile 0.9% NaCl was added to all wells before cell density (OD_600_) and GFP fluorescence (excitation wavelength, 485 nm; emission wavelength, 535 nm) were measured for the wells in each plate. The ability of each compound to reduce the c-di-GMP level was then evaluated by comparing the specific GFP (GFP/OD_600_) output for the compounds in comparison to the controls. Compounds that affected growth, evaluated from continuous OD_600_ measurements, were excluded from further validation.

Compounds that reduced the GFP output with 30% or more were tested again to confirm their activity, using the same setup as described above. To further confirm activity, compounds that reduced the GFP output >40% in both screenings, were validated by continuous readings of the OD_600_ and GFP output for 24 h, using a 96-well microtiter plate setup as follows. In 96-well microtiter plates, an overnight culture of the monitor c-di-GMP strain was diluted 100-fold into 100 μl ABTrace media aliquots^68^ supplemented with 0.2% glucose, 0.5% casa amino acids, 1% DMSO, 60 μg/ml gentamicin, 1 µM FeCl_3,_ and, respectively, 100 µM, 50 µM, 25 µM, 12 µM, and 0 µM of each compound. The microtiter plates were incubated at 37°C at 440 RPM in a TECAN reader (Infinite F200 PRO), and corresponding values of cell density (OD_600_) and GFP fluorescence were measured every 20 min for 24 h.

### Determination of c-di-GMP concentrations in *P. aeruginosa* by HPLC coupled MS-MS

C-di-GMP levels in *P. aeruginosa* were determined by HPLC coupled MS-MS quantification of cell extracts. To quantify the c-di-GMP-reducing potency of H6-335 and H6-335-P1, an overnight culture of *P. aeruginosa* Δ*wspF*Δ*pel*Δ*psl* was diluted 100-fold into 25 ml of ABTrace medium^68^ supplemented with 0.2% glucose, 0.5% cas amino acids, 60 μg/ml gentamicin, 1 µM FeCl_3_, 0.05% DMSO, and either 100 µM H6-335, 100 µM H6-335-P1, or no compound as control. Serving as a control for low c-di-GMP levels, an overnight culture of strain *P. aeruginosa* Δ*wspF*Δ*pel*Δ*psl*/pYhjH^G^ (carrying a plasmid encoding a constitutively expressed YhjH PDE) was also diluted 100-fold into 25 ml of the ABTrace medium. The four cultures were incubated at 37°C on a rotary shaker at 200 RPM and following 8 h of growth (at the entry into stationary phase) culture samples for c-di-GMP extraction and protein quantification were collected from each of the cultures. Subsequently, c-di-GMP extracts were prepared and c-di-GMP levels were quantified by HPLC coupled MS-MS as previously described^27^. Protein quantifications were carried out using the Pierce 660 nm Protein Assay (Thermo Scientific Cat. No 22660) according to the manufacturer’s protocol.

### Gauging of the c-di-GMP content in *P. aeruginosa* c-di-GMP reporter strains exposed to H6-335 or H6-335-P1

To evaluate the impact of H6-335 or H6-335-P1 on the c-di-GMP level of the c-di-GMP reporter strains *P. aeruginosa* Δ*wspF*Δ*pel*Δ*psl*/pCdrA-gfp and *P. aeruginosa* Δ*bifA*Δ*wspF*Δ*pel*Δ*psl*/pCdrA-gfp, 20 h old cultures of the respective strains were diluted 100-fold in microtiter plate wells (Nunc) containing 100 µl aliquots of ABTrace medium^68^ supplemented with 0.2% glucose, 0.5% Cas amino acids, 60 µg/ml gentamicin, 1 µM FeCl_3_, 1% DMSO, and concentrations of H6-335 or H6-335-P1 as indicated in the text. Subsequently the microtiter plates were incubated at 37°C and 440 RPM in a TECAN reader (Infinite F200 PRO), and corresponding values of cell density (OD_600_) and GFP fluorescence were measured every 20 min for 24 h.

### Microtiter plate-based biofilm assay

The effect of H6-335-P1 on biofilm development of various *P. aeruginosa* strains (specified in the text) was evaluated using a 96-well microtiter plate-based biofilm assay as described by Groizeleau et al.^27^. In brief, a *P. aeruginosa* overnight culture was diluted 10^4^ times in microtiter plate wells (Nunc) containing 100 μl ABTrace medium aliquots^68^ supplemented with 0.2% glucose, 0.5% cas amino acids, 1 µM FeCl_3_, 0.2% DMSO, and either 100 µM, 50 µM, 25 µM, 12 µM, 6 µM, or 0 µM of H6-335-P1. Then, the microtiter plate was sealed with an air permeable lid (CR1596, Enzyscreen, Netherlands), and incubated on a rotary shaker at 160 RPM and 37°C. Following 10 h of incubation, the culture supernatants were discarded and the biofilms in each well were stained with crystal violet. Finally, the biofilm biomass was quantified by resuspending the stained biofilms into 300 µl of 30% acid acetic, followed by OD_590_ readings using a VIKTOR plate reader (Perkin Elmer).

For measuring dispersal, a *P. aeruginosa* PAO1 overnight culture was diluted to an OD_600_ of 0.1 in ABTrace medium^68^ supplemented with 0.5% glucose, 0.5% Cas amino acids, and 1 µM FeCl_3_. Aliquots of 490 μl of the diluted culture were then transferred to the wells of 48-well microtiter trays. Subsequently, the microtiter trays were incubated for 18 h at 37°C on a rotary shaker (180 RPM) to allow biofilms to form in the wells. Then, 10 μl medium containing H6-335-P1 or DMSO-control (final concentration of 25 μM H6-335-P1/1% DMSO) was added to the wells. Finally, samples were withdrawn from the liquid medium at various time-points and plated for CFU determinations.

Dispersal of PDE mutant biofilms was evaluated using a 96-well microtiter plate-based biofilm assay. Overnight cultures of *P. aeruginosa* wild type and PDE mutants were grown in ABTrace medium^68^ supplemented with 0.5% glucose, 0.5% cas amino acid, and 1 µM FeCl_3_ and diluted 1:100 into fresh medium. The diluted overnight cultures were transferred to 96-well microtiter plates with 190 μl culture per well and incubated at 37°C on a rotary shaker (180 RPM) to allow biofilm formation in the wells. The biofilms were grown for 18 h, at which time point either 10 µl of 2 mM H6-335-P1 (in 20% DMSO) or 10 µl of 20% DMSO (final concentration of 100 μM H6-335-P1/1% DMSO) were added to each well. After 2 subsequent hours of incubation, the culture supernatants were discarded and the wells were washed with 0.9% NaCl. The amounts of biofilm present in the wells were then quantified by crystal violet staining^27^. PDE mutants were obtained from the *P. aeruginosa* PAO1 transposon mutant collection of the University of Washington.

### Construction of *bif*A deletion mutants

Knockout of the *bif*A gene in the *P. aeruginosa* PAO1 wild type, *ΔwspF* and *ΔwspFΔpelΔpsl* strains was done using the knockout plasmid pK18-*bif*A-del^69^, and a modified version of the gene knockout protocol outlined in the study of An and coworkers^69^. In brief, an 18 h old culture of the *P. aeruginosa* strain (propagated in LB at 37°C) was diluted twofold into 42°C warm LB medium, and kept at 42°C for 4 h. Then a mating solution containing a 1:1 mixture of heat-treated *P. aeruginosa* culture and a late exponential culture of the donor strain *E. coli* S17-1/pK18-bifA-del was prepared, and subsequently spotted onto a LB plate. Following 20 h of mating at 30°C, the resulting mating spot was resuspended in 1 ml of 0.9% NaCl, and transconjugants (merodiploids) were selected on ABTrace plates supplemented with 10 mM sodium citrate, 1 µM FeCl_3_, and 60 µg/ml gentamicin. Next, merodiploids were subjected to SacB-based counter selection by means of repeated streaking onto both LB plates supplemented with 60 µg/ml gentamicin and No-NaCl-LB plates supplemented with 10% sucrose. Sucrose selection at 30°C was repeated until the emergence of sucrose resistant, gentamicin-sensitive colonies, to obtain double cross-over transconjugants. Finally, PCR analysis with the *bif*A-flanking primer pair *bif*A-D-up-F-*Eco*RI/*bif*A-D-Dn-R-*Hind*III was conducted on sucrose resistant, gentamicin-sensitive transconjugants, and double cross-over transconjugants carrying the mutant *bifA* allele was identified and selected for further analysis. The generated *bif*A deletion mutants with a 1380 bp internal deletion in the *bifA* gene were verified by DNA sequencing.

### Construction of a *P. aeruginosa Δbif*A*ΔwspF* strain with chromosomal insertion of a P_BAD_-*bif*A fusion

The *ara*C-P_BAD_-*bif*A expression cassette of plasmid pJBAMG13^11^ was inserted into the chromosome of *P. aeruginosa Δbif*A*ΔwspF* using the protocol described by Andersen et al.^11^. Initially, a transconjugant with plasmid pJBAMG13 inserted into the chromosomal ϕCTX *attB* site was constructed by three-parental mating using *E. coli* DH5-α/pJBAMG13 as donor, *E. coli* HB101/pRK600 as helper, and *P. aeruginosa Δbif*A*ΔwspF* as recipient. Then, the transconjugant was transformed with plasmid pFLP2 encoding Flp recombinase^70^ to obtain a pFLP2 transformant where the Fret flanked plasmid backbone of pJBAMG13 had been excised. Subsequently, the *P. aeruginosa Δbif*A*ΔwspF* strain containing one chromosomal copy of the *ara*C-P_BAD_-*bif*A expression cassette was cured for plasmid pFLP2 using sucrose selection. Finally, the chromosomal location of the *ara*C-P_BAD_-*bif*A expression cassette in strain *P. aeruginosa Δbif*A*ΔwspF* was verified by PCR using the primer pair Pser-up/Pser-down^70^.

### Construction of plasmid pYhjH^G^

To obtain a gentamicin resistance-encoding *P. aeruginosa* expression vector with constitutive expression of the *E. coli yhjH* PDE gene, the *yhjH* expression cassette of pYhjH^9^ was cloned into the broad host range plasmid pBBR1MCS5^71^. In brief, the *yhjH* expression cassette was excised from plasmid pYhjH by restriction with *Not*I, and then ligated to partially *Not*I-digested pBBR1MCS5 vector. The resulting ligation was electroporated into *E. coli* DH5α and transformants were selected on LB plates supplemented with 10 µg/ml gentamicin and 10 µg/ml chloramphenicol. Restriction analysis performed on plasmids isolated from selected transformants revealed that all four possible plasmid structure combinations were represented in the plasmid collection (pBBR1MCS5 contains two *Not*I sites, and the 2.1 Kb *Not*I expression cassette can be inserted in two orientations). Among these four plasmid variants, the variant where the *yhjH* expression cassette had been inserted into the *Not*I site located in the MCS of pBBR1MCS5, and where the *yhjH* gene is transcribed in the same orientation as the gentamicin resistance gene, was selected and designated pYhjH^G^. Finally, pYhjH^G^ was electroporated into *P. aeruginosa ΔwspFΔpslΔpel*. *P. aeruginosa* containing pYhjH^G^ could be grown with selection for plasmid maintenance without obvious growth defects, in contrast to *P. aeruginosa* containing pYhjH.

### Transposon mutagenesis

Mariner transposon mutagenesis in the *P. aeruginosa ΔwspF* strain was done using the protocol of Kulasekara^72^. In brief, following a 2 h biparental mating between *P. aeruginosa ΔwspF* and *E. coli* S17-λ*pir*/pBT20 on LB plates, the conjugation spots were collected and resuspended in 0.9% NaCl. Then 32 × 200 µl aliquots of the conjugation mixture were spread onto 15 cm wide LB plates supplemented with 1% agar, 20 µg/ml coomassie blue, 40 µg/ml CR, 60 µg/ml gentamicin, and 100 µM H6-335-P1. The plates were subsequently incubated at 37°C for 2 days, which resulted in the formation of ~32,000 transposon mutant colonies. The parental *P. aeruginosa ΔwspF* strain forms white colonies on agar plates with CR and H6-335-P1, whereas mutants that produce a large amount of polysaccharide despite the presence of H6-335-P1 form red colonies on plates with CR and H6-335-P1. The chromosomal insertion site of the mariner transposon in selected mutants was identified by sequencing, using the two-step arbitrary PCR protocol described by Kulasekara^72^.

### Flow-cell biofilm experiments

The flow-cell system was assembled and run as described by Crusz et al.^73^. Biofilms were grown at 37°C in continuous-culture, once-through, three-channel flow-chambers (individual channel dimensions of 1 × 4 × 40 mm) perfused with sterile ABTrace minimal medium^68^ supplemented with 0.3 mM glucose. Growth medium supplemented with 25 µM H6-335-P1 was used for treatments, whereas medium supplemented with 0.025% (v/v) DMSO was used as control to account for effects of the DMSO vehicle used to dissolve H6-335-P1. *P. aeruginosa* PAO1 tagged with miniTn7-*gfp* at a neutral chromosomal locus was used for the experiments. Each channel was inoculated with 300 µl of overnight culture diluted to an OD_600_ of 0.005 in 0.9% (w/v) NaCl, and after a static attachment phase of 1 h, the flow of medium was initiated to allow the biofilms to develop. For experiments investigating the effect of H6-335-P1 on biofilm formation, treatment was ensued from the onset of the experiment and the biofilms were visualized after 24 h and 48 h of growth. For experiments investigating the dispersal effect of H6-335-P1, biofilms were allowed to develop for 48 h in flow-cells irrigated with DMSO-control medium before being challenged with a medium containing H6-335-P1. Treatment was conducted at room temperature and the dispersal effect on the biofilms was visualized after 4 h. Visualization of the biofilms by confocal laser scanning microscopy was performed with a Zeiss LSM710 confocal laser scanning microscope (Carl Zeiss, Germany) set for detecting GFP fluorescence (excitation: 488 nm; emission: >517 nm), and using a ×63/1.4 oil objective. Simulated fluorescence projections in 3D were generated from the image stacks using the IMARIS software package (Bitplane, Oxford Imaging, UK), and the resulting images were processed for publication using Photoshop (Adobe, USA).

### RNA preparation for qRT-PCR and RNA-seq

The *P. aeruginosa* PAO1 wild type and *P. aeruginosa* Δ*wspF*Δ*pel*Δ*psl* mutant were grown at 37°C and 200 RPM in ABTrace medium^68^ supplemented with 0.2% glucose, 0.5% cas amino acids, and 1 µM FeCl_3_, and at an OD_600_ of 0.5 the cultures were diluted to OD_600_ 0.01, and incubation was continued. When an OD_600_ of 0.5 was reached again, each culture was divided in two. For both the wild type and mutant, one culture was supplemented with 50 µM of H6-335-P1 dissolved in DMSO, whereas the other culture was supplemented with an equal concentration of DMSO as control. Samples were retrieved at mid-log, early stationary, and stationary growth phase after 1.5, 2.5, and 3.5 h of further incubation. Samples were centrifuged at 10,000 × *g*, and were resuspended in two volumes of RNA protect Bacteria Reagent (Qiagen). After 5 min incubation at room temperature, the cells were centrifuged at 10,000 × *g* and the supernatant was discarded. The cells were frozen at -80°C until further analysis. Isolation of RNA was performed with RNeasy minipurification kit (Qiagen) with DNase I treatment according to the manufacturer’s guidelines. Subsequently, synthesis of cDNA was done from 1 μg RNA using a High Capacity RNA-to-cDNA kit (Applied Biosystems). Quality control of RNA samples for purity and integrity was done by NanoDrop spectrophotometer (Thermo Scientific) measurements, Agilent 2100 Bioanalyzer (Agilent Technologies), and visual inspection of samples after agarose gel electrophoresis. RNA was purified from three replicate set of cultures for RNA-seq and qRT-PCR.

### RNA-seq and data analysis

Assessment of global gene expression was done by Illumina RNA-seq. RNA-seq was conducted for three biological replicates of each condition. Ribosomal RNA was removed using Ribopool 2 targeting *P. aeruginosa* rRNAs (SiTOOLs Biotech). Libraries were produced using the NEBNEXT Ultra II RNA Library Prep (New England Biolabs), and were sequenced using the Illumina HiSeq2500 platform with paired-end protocol and 150 nucleotide read lengths. Sequencing adapters were removed and reads were filtered (length > 10nt) with cutadapt v2.4. Trimmed and filtered reads where then aligned to the *Pseudomonas aeruginosa* reference genome (NCBI Refseq: GCA_000006765.1) using bowtie2 v2.3.4.1 with default settings for paired-end reads. Read-pairs mapping to gene-level features were then counted with the featureCounts function of Subread v1.6.2 using default settings. Analysis of differential gene expression was performed with the R package, DESeq2 v.1.26.0. A “condition” metadata group was created, which uniquely grouped each set of three biological replicates. This was used for the “design” DESeq2 argument. All samples included in this model. Results of the differential gene expression analyses were then extracted for comparisons of interest with the “results()” function. A table of normalized read counts was also created using the vst() function with blind=FALSE.

### qRT-PCR

For quantitative real-time PCR, amplification was performed with Power Up Amplification SYBR green Master Mix in a StepOne Plus thermal cycler (Applied Biosystems). Forty cycles were run with the following settings: initial denaturation at 95°C for 10 min, annealing and extension at 60°C for 1 min. A no-template control was included in every run for every primer pair. The genes *rpoD*, *rpoS,* and *oprL* were used as endogenous controls. Sequences of the primers that were used are shown in Supplementary Table 3. To evaluate PCR efficiency a standard curve with 10-fold dilutions of cDNA was generated for every primer pair. Two technical replicates were used for every biological replicate.

### Antimicrobial killing assays in vitro

An overnight culture of *P. aeruginosa* PAO1 was grown in ABTrace medium^68^ supplemented with 0.5% glucose, 0.5% cas amino acids, and 1 µM FeCl_3_, and was diluted 1:1000 into fresh medium. Cultures arranged in triplicates were grown in microtiter plates with 125 µl medium per well, with agitation (110 RPM) for 24 h at 37°C, in order for biofilms to form on polystyrene pegs (Nunc-TSP) that were submerged in the wells. Biofilms on the pegs were washed twice in 0.9% NaCl and were incubated for 2 h with either 100 µM H6-335-P1 or DMSO-control.

Antibiotic-mediated killing of dispersed bacteria was assayed as follows: after 2 h of incubation with 100 µM H6-335-P1 or DMSO-control, the planktonic cultures were transferred to a new microtiter plate, and medium supplemented with either tobramycin or ciprofloxacin (and 100 µM H6-335-P1 or DMSO-control) was added, resulting in final concentrations of 30 µg tobramycin/ml and 0.5 µg ciprofloxacin/ml. The plates were incubated at 37°C. From each well, 20 µl aliquots were withdrawn every hour for 4 h, and were diluted in 10-fold steps and spread on agar plates, for CFU determination.

Determination of antibiotic-mediated killing for biofilm cells was done as follows: After 2 h of incubation with 100 µM H6-335-P1 or DMSO-control, the biofilms on the pegs were washed once in 0.9% NaCl and were challenged with 30 µg/ml tobramycin or 0.5 µg/ml ciprofloxacin for 8 h at 37°C. The H6-335-P1-treated biofilms received H6-335-P1 along with the antibiotics, whereas the DMSO-control biofilms received DMSO along with the antibiotics. Subsequently, samples were assayed every two hours for 8 h. For this purpose, the biofilms were washed twice in 0.9% NaCl, and were then disrupted by five minutes of degassing and 3 × 10 min sonication in an ultrasonic bath. To determine cell viability, the suspensions of disrupted cells were diluted in 0.9% NaCl in 10-fold steps, and 20 µl of each dilution was spotted on LB-agar plates. CFUs were determined after overnight incubation at 37°C.

### In vivo implant biofilm infection model

Animal experiments were performed with the wild-type *P. aeruginosa* strain (PAO1) obtained from Professor Barbara Iglewski (University of Rochester Medical Center, NY, USA).

Female BALB/c mice were purchased from either Taconic M&B A/S (Ry, Denmark) or Janvier Labs (Saint-Berthevin, France) at 8–9 weeks of age. Mice were maintained on standard mouse chow and water ad libitum for 1–2 weeks before insertion of implants. The animal studies were carried out in accordance with the European convention and Directive for the Protection of Vertebrate Animals used for Experimental and Other Scientific Purposes, and the Danish law on animal experimentation. All experiments were authorized and approved by the National Animal Ethics Committee of Denmark (The Animal Experiments Inspectorate, dyreforsoegstilsynet.dk), and given the permit numbers 2014-15-0201-00259 and 2018-15-0201-01389.

Silicone implants were prepared as described by Christensen et al.^74^. In short, sterile silicone implants (4 mm long, 6 mm OD × 4 mm ID) were incubated with *P. aeruginosa* cultures (resuspended overnight culture in 0.9% NaCl with an OD_600_ of 0.1) for 20 h for bacterial adhesion to the implant. Animals were challenged according to the method described in Christensen et al.^75^. Mice were anesthetized using subcutaneous (sc) injections in the groin area with hypnorm/midazolam (Roche) (one part hypnorm (0.315 mg/ml fentanyl citrate and 10 mg/ml fluanisone), one part midazolam (5 mg/ml), and two parts sterile water). For post-operative pain, mice were given one drop of bupivacaine (5 mg/ml) using an 18 G needle at the incision site. In addition, mice received injections of 0.1 μg buprenorphinum per gram bodyweight sc every 8th hours the first 24 h PI. At the termination of the experiments, the mice were euthanized by intraperitoneal (ip) injection of 10.0 μl per gram bodyweight Pentobarbital (200 mg/ml) with lidocaine hydrochloride (20 mg/ml) (DAK).

For all experiments, mice had the implants inserted in the peritoneal cavity at time *t* = 0. Treatments with H6-335-P1 were administered as ip injections at times *t* = 24 h and *t* = 26 h PI. H6-335-P1 was dissolved in a 20% 2-hydroxypropyl-β-cyclodextrin solution (vehicle) to prepare stock-solutions of 0.6 mM to 2.5 mM H6-335-P1, of which the mice were administered 0.2 ml dosages. The mice had an average bodyweight of 20 g, considered as 20 ml body volume per mouse, which roughly corresponds to dosages of 1 to 6 μg H6-335-P1 compound per gram bodyweight, or 6–25 μM H6-335-P1.

Tobramycin sulfate was dissolved in 0.9% NaCl to a concentration of 3 g tobramycin/l. Mice were injected ip with 30 μg tobramycin per gram bodyweight one time at 26 h PI. Ciprofloxacin was dissolved in 0.9 % NaCl and adjusted to pH 6.8 with HCl, to a concentration of 1 g ciprofloxacin/l. Mice were injected ip with 10 μg ciprofloxacin per gram bodyweight one time at 24 h PI.

At 28 h PI, the mice were euthanized, and the implants were retrieved in order to determine CFU per implant. After silicone implants were removed from the mice, they were placed in centrifuge tubes containing 2 ml cold 0.9% NaCl and kept on ice. Shortly after, the tubes were placed in an ultrasound bath (Bransonic® model 2510, Branson Ultrasonic Corporation, USA) for 10 min (5 min degas followed by 5 min sonic). The saline bacteria suspensions were then serially diluted and plated on blue agar plates (State Serum Institute, Denmark), which are selective for Gram-negative bacteria. The plates were incubated at 37°C overnight before the determination of CFU per implant.

### Statistical information

Statistical significance was evaluated by one-way or two-way analysis of variance, with Sidak’s post-tests for experiments with multiple comparisons. To compare the bacterial counts (CFU) between two groups of mice, the Mann–Whitney *U* test was used (analysis of non-parametric data). Sample sizes for the mice experiments were chosen based on the number of mice required to reach statistical significance using the Mann–Whitney test from previous experiments using the biofilm infection model. The statistical program GraphPad Prism was used for calculating *P* values, and *P* values ≤ 0.05 were considered significant. Sample size (*n*) and statistical methods are specified in the figure legends. The *P* value of the transcriptomic analysis was calculated from DESeq2 with the Wald test followed by Benjamini-Hochberg adjustment for multiple comparisons with a false-discovery rate of 0.1.

## Supplementary information

Supplementary Information

## Data Availability

The data that support the findings of this study are available from the corresponding author upon reasonable request. The RNA-Seq data have been deposited in the NCBI Sequence Read Archive (SRA) database with accession code PRJNA673995.
