# Supplementary material for: Identification of small molecules that interfere with c-di-GMP signaling and induce dispersal of Pseudomonas aeruginosa biofilms
Source: NPJ Biofilms Microbiomes. 2021 Jul 9;7:59. doi: 10.1038/s41522-021-00225-4 (PMC8271024; doi:10.1038/s41522-021-00225-4)
Supplement: Supplementary file 1 — Supplementary Information [file 41522_2021_225_MOESM1_ESM.pdf]

Supplementary Information

Identification of small molecules that interfere with c-di-GMP signaling and induce dispersal of *Pseudomonas aeruginosa* biofilms

Jens Bo Andersen[1], Louise Dahl Hultqvist[1], Charlotte Uldahl Jansen[2], Tim Holm Jakobsen[1], Martin Nilsson[1], Morten Rybtke[1], Jesper Uhd[2], Blaine Gabriel Fritz[1], Roland Seifert[3], Jens Berthelsen[1], Thomas Eiland Nielsen[1,4], Katrine Qvortrup[2], Michael Givskov[1,4¤], Tim Tolker-Nielsen[1]*

[1]Costerton Biofilm Center. Department of Immunology and Microbiology, Faculty of Health and Medical Sciences, University of Copenhagen, DK-2200 Copenhagen, Denmark.
[2]Department of Chemistry, Technical University of Denmark, DK-2800 Lyngby, Denmark.
[3]Institute of Pharmacology and Research Core Unit Metabolomics, Hannover Medical School Carl-Neuberg-Straße 1, D-30625 Hannover, Germany.
[4]Singapore Centre for Environmental Life Sciences Engineering, Nanyang Technological University, Singapore.

Supplementary Tables

Supplementary Table 1. Strain list.

Strains	Description	Reference
E.coli :		
DH5-α	F^- *endA1 glnV44 thi-1 recA1 relA1 gyrA96 deoR nupG purB20 φ80dlacZΔM15 Δ(lacZYA-argF)*U169, hsdR17(r_k^- m_k^+), λ^-	Lab collection
HB101	*rec*A *thi pro leu hsd*RM^+, Sm^R	Kessler et al. 1992
RK600	HB101 carrying pRK600	Kessler et. al. 1992
S17-1 λ*pir*	Str^R, Tet^R, F- RP4-2-Tc::Mu *aph*A::Tn7 *rec*A λpir lysogen	Lab collection
S17-1	*recA pro* (RP4-2Tet::Mu Kan::Tn*7*)	Simon et al. 1983
AR3110	Cellulose producing strain	Serra et al. 2013
P. aeruginosa:		
PAO1	*Pseudomonas aeruginosa* reference strain	Stover et al. 2000
PAO1::*Tn*7-*gfp*	PAO1 chromosomally tagged with *gfp*	Andersen et al. 2021
Δ*wspF*	*wspF* deletion mutant of PAO1	Rybtke et al. 2012
Δ*bifA*	*bifA* deletion mutant of PAO1	This study
Δ*bifA*Δ*wspF*	*wspF, bifA* double deletion mutant of PAO1	This study
ΔwspFΔpelΔpsl	*wspF*, *pel*, *psl* triple deletion mutant of PAO1	Rybtke et al. 2012
ΔwspFΔpelΔpsl/pCdrA-gfp	*ΔwspFΔpelΔpsl* carrying plasmid pCdrA-gfp	Rybtke et al. 2012
ΔbifAΔwspFΔpelΔpsl	*bifA, wspF*, *pel*, *psl* quadruple deletion mutant of PAO1	This study
ΔbifAΔwspFΔpelΔpsl/pCdrA-gfp	*ΔbifAΔwspFΔpelΔpsl* carrying plasmid pCdrA-gfp	This study
ΔbdlAΔwspFΔpelΔpsl/pCdrA-gfp	*ΔbdlAΔwspFΔpelΔpsl* carrying plasmid pCdrA-gfp	This study
ΔsiaDΔwspFΔpelΔpsl/pCdrA-gfp	*ΔsiaDΔwspFΔpelΔpsl* carrying plasmid pCdrA-gfp	This study
ΔwspFΔpelΔpsl/pYhjH^G	*ΔwspFΔpelΔpsl* carrying plasmid pYhjH^G	This study
Δ*bifA*Δ*wspF* P_{BAD}-*bifA*	Δ*bifA*Δ*wspF* with chromosomal insertion of *ara*C-P_{BAD}-*bif*A	This study
PAO1(Iglewski)	PAO1 with reduced production of C4-HSL (Köhler et al. 2001)	Obtained from B. Iglewski (University of Rochester Medical Center, NY, USA)
MPAO1	*P. aeruginosa* PAO1 from Dr. Manoils laboratory.	Jacobs et al. 2003, Held et al. 2012
MPAO1(PW1520)	PA0285-H01::ISlacZ/hah	Jacobs et al. 2003, Held et

		al. 2012
MPAO1(PW4747)	PA2200-F11::ISlacZ/hah	Jacobs et al. 2003, Held et al. 2012
MPAO1(PW5718)	arr(PA2818)-A01::ISlacZ/hah	Jacobs et al. 2003, Held et al. 2012
MPAO1(PW7454)	PA3825-G08::ISphoA/hah	Jacobs et al. 2003, Held et al. 2012
MPAO1(PW5308)	PA2567-G11::ISlacZ/hah	Jacobs et al. 2003, Held et al. 2012
MPAO1(PW9918)	PA5295-G02::ISlacZ/hah	Jacobs et al. 2003, Held et al. 2012
MPAO1(PW7948)	PA4108-B01::ISlacZ/hah	Jacobs et al. 2003, Held et al. 2012
MPAO1(PW7674)	rocR(PA3947)-E06::ISphoA/hah	Jacobs et al. 2003, Held et al. 2012
MPAO1(PW4043)	mucR(PA1727)-C01::ISlacZ/hah	Jacobs et al. 2003, Held et al. 2012
MPAO1(PW8371)	bifA(PA4367)-D09::ISlacZ/hah	Jacobs et al. 2003, Held et al. 2012
MPAO1(PW8754)	morA(PA4601)-C02::ISlacZ/hah	Jacobs et al. 2003, Held et al. 2012
MPAO1(PW2569)	rdbA(PA0861)-F02::ISlacZ/hah	Jacobs et al. 2003, Held et al. 2012
MPAO1(PW6567)	nbdA(PA3311)-B12::ISlacZ/hah	Jacobs et al. 2003, Held et al. 2012
MPAO1(PW9424)	dipA(PA5017)-A01::ISphoA/hah	Jacobs et al. 2003, Held et al. 2012
MPAO1(PW2060)	PA0575-A03::ISphoA/hah	Jacobs et al. 2003, Held et al. 2012
MPAO1(PW3134)	PA1181-H12::ISphoA/hah	Jacobs et al. 2003, Held et al. 2012
MPAO1(PW3602)	PA1433-B09::ISlacZ/hah	Jacobs et al. 2003, Held et al. 2012
MPAO1(PW4568)	PA2072-B02::ISlacZ/hah	Jacobs et al. 2003, Held et al. 2012
MPAO1(PW6465)	PA3258-D03::ISphoA/hah	Jacobs et al. 2003, Held et al. 2012
MPAO1(PW9346)	fimX(PA4959)-F01::ISlacZ/hah	Jacobs et al. 2003, Held et al. 2012
MPAO1(PW10193)	PA5442-C04::ISlacZ/hah	Jacobs et al. 2003, Held et al. 2012
MPAO1(PW4665)	PA2133-B04::ISlacZ/hah	Jacobs et al. 2003, Held et al. 2012
MPAO1(PW2283)	PA0707-E04::ISlacZ/hah	Jacobs et al. 2003, Held et al. 2012.
MPAO1(PW5317)	PA2572-D10::ISlacZ/hah	Jacobs et al. 2003, Held et al. 2012.
MPAO1(PW9032)	PA4781-E02::ISlacZ/hah	Jacobs et al. 2003, Held et al. 2012.

Supplementary Table 2. Plasmid list.

Plasmid	Description	Reference
pK18GT-bifA-del	*bif*A deletion vector, Gm^R	An et al. 2010
pJBAMG13	*ara*C-P_{BAD}-*bif*A fusion of pJBAMG10 inserted into the BP clonase sites of pminiCTX2T2.1-GW	Andersen et al. 2021
pBT20	Transposon delivery vector	Kulasekara 2014
pJN105	Arabinose inducible expression vector	Newman & Fuqua 1999
pJN105::bifA	Plasmid for arabinose induction of *bifA*. Also termed pJBAMG10.	Andersen et al. 2021
pCdrA-gfp	pUCP22Not-P_{cdrA}-*gfp*(mut3)-T_0-T_1, Amp^R, Gm^R	Rybtke et al. 2012
pFlp2	Source of Flp2 recombinase, Amp^R	Hoang et al. 1998
pRK600	Mobilization plasmid, Cm^R	Kessler et al. 1992
pQE30	Expression vector of N-terminally His-tagged proteins Amp^R	Qiagen
pQE30::bifA180	Plasmid for IPTG-inducible expression of BifA180. A *bifA*180 fragment was PCR amplified from *P. aeruginosa* DNA with primers Bif180-*Sac*I and Bif180-*Hind*III, and cloned in the *Sac*I and *Hind*III site of pQE30.	
pYjhH	*yhjH* gene of *E. coli* cloned in pBBR1MCS3, Tet^R, Cm^R	Gjermansen et al. 2006
pBBR1MCS5	Broad host range plasmid, Gm^R, Cm^R	Kovach et al. 1995
$pYhjH^G$	*yhjH* expression cassette of pYhjH cloned into plasmid pBBR1MCS5, Gm^R	This study

Supplementary Table 3. Primer list

Primers	Description	References
rpoD fwd	5'-ACAAGATCCGCAAGGTACTGAAG-3'	Jakobsen et al. 2012
rpoD rev	5'-CGCCCAGGTGCGAATC-3'	Jakobsen et al. 2012
rpoS fwd	5'-CTCCCCGGGCAACTCCAAAAG-3'	This study
rpos rev	5'-CGATCATCCGCTTCCGACCAG-3'	This study
oprL fwd	5'-ATGGAAATGCTGAAATTCGGC-3'	This study
oprl rev	5'-ACCTTCACCGGAAGCATCG-3'	This study
bifA fwd	5'-CATCTTCATCTCCGGCATTCTC-3'	This study
bifA rev	5'-TTGGTCAGCATCCAGTGGTAGA-3'	This study
cdrA fwd	5'-CAACAGTCAGTTCAACGACCTC-3'	This study
cdrA rev	5'-AACGCTGGCTGAAATACTCG-3'	This study
*bif*A-D-Up-F-*Eco*RI	5'-GGAATTCTGCTGACCTGCGACGTCTGGGAAC-3'	An et al. 2010
*bif*A-D-Dn-R-*Hind*III	5'-CCCAAGCTTGTGCGGTGATCCGTGAATGGAAGG-3'	An et al. 2010
Pser-up	5'-CGAGTGGTTTAAGGCAACGGTCTTGA-3'	Hoang et al. 2000
Pser-down	5'-AGTTCGGCCTGGTGGAACAACTCG-3'	Hoang et al. 2000

TnMseq	5'-CACCCAGCTTTCTTGTACAC-3'	Rybtke et al. 2015
Rnd1-TnM	5'-GTGAGCGGATAACAATTTCACACAG-3'	Rybtke et al. 2015
Rnd1-pP1	5'-GGCCACGCGTCGACTAGTCANNNNNNNNNNGAT AT-3'	Rybtke et al. 2015
Rnd1-pP2	5'-GGCCACGCGTCGACTAGTCANNNNNNNNNNACG CC-3'	Rybtke et al. 2015
Rnd2-TnM	5'- ACAGGAAACAGGACTCTAGAGG-3' ??	Rybtke et al. 2015
Rnd2-Pp	5'- GGCCACGCGTCGACTAGTAC-3' ??	Rybtke et al. 2015
BifA180-*Sac*I	5'- GGCGGAGCTCCACTGGATGCTGACCAAGCC	This study
BifA180-*Hind*III	5'- GGCGAAGCTTTCAGGGCCGTTCGCTGCTGGTGG	This study

Supplementary Figures

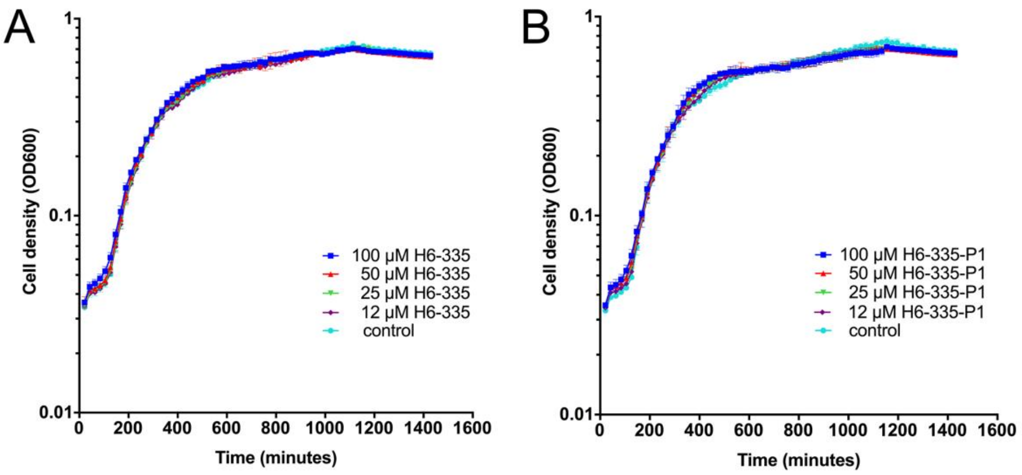

Supplementary Figure 1. Effects of H6-335 **(A)** and H6-335-P1 **(B)** on the growth of the c-di-GMP monitor strain *P. aeruginosa* Δ*wspF*Δ*pel*Δ*psl/*pCdrA-gf*p.* Bacteria where grown in the wells of microtiter plates in the presence of various concentrations of H6-335 or H6-335-P1. Cell density (OD_{600}) was measured every 20 minutes for 24 hours. Cell densities are plotted as a function of time and H6-335 or H6-335-P1 concentration. Mean and standard deviation (bars) of 3 biological replicates (n=3) are shown.

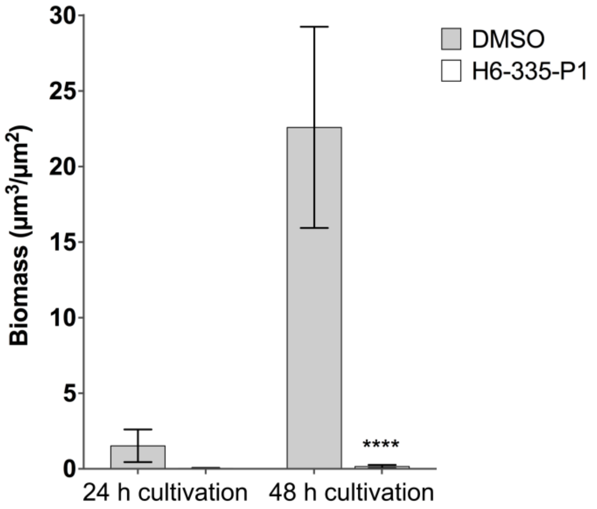

Supplementary Figure 2. H6-335-P1-mediated inhibition of *P. aeruginosa* biofilm formation in flow-cells. *P. aeruginosa* was cultivated in flow-cells perfused with growth medium with or without 25 μM H6-336-P1. CLSM micrographs of the adherent bacteria were acquired after 24 and 48 hours of cultivation, and the amount of bacterial biomass in the flow-cells was quantified by the use of COMSTAT image analysis (Heydorn et al., 2000). Average biomass calculated from three independent biofilms is shown. Error bars indicate standard deviations. One-way ANOVA analysis was used to calculate significance values (****, p<0.0001)

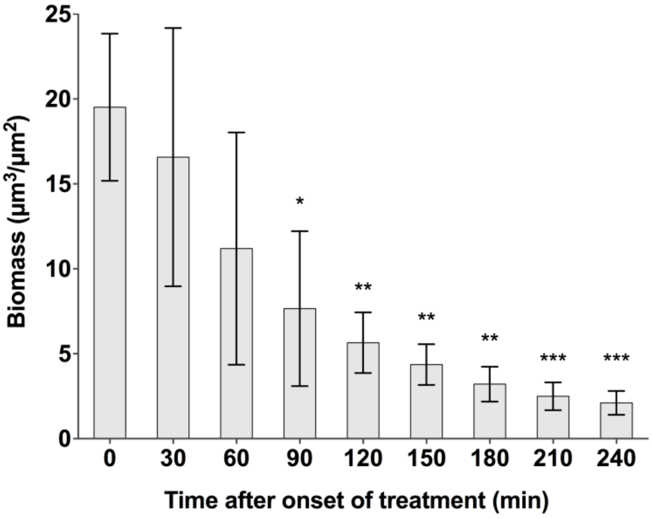

Supplementary Figure 3. H6-335-P1 mediated dispersal of *P. aeruginosa* biofilms in flow-cells. *P. aeruginosa* biofilms were cultivated in flow-cells irrigated with growth medium supplemented with 0.025% DMSO. After 48 hours of cultivation the flow-through medium was shifted so that it contained 0.025% DMSO and 25 μM H6-335-P1. CLSM micrographs were acquired immediately before the introduction of H6-336-P1 and at 30 minutes intervals after the introduction of the compound. The amount of bacterial biomass in the flow-cells was quantified by the use of COMSTAT image analysis (Heydorn et al., 2000). Average biomass calculated from three independent biofilms is shown. Error bars indicate standard deviations. One-way ANOVA analysis was used to calculate significance values compared to time zero (*, p<0.05; **, p<0.01; ***, p<0.001).

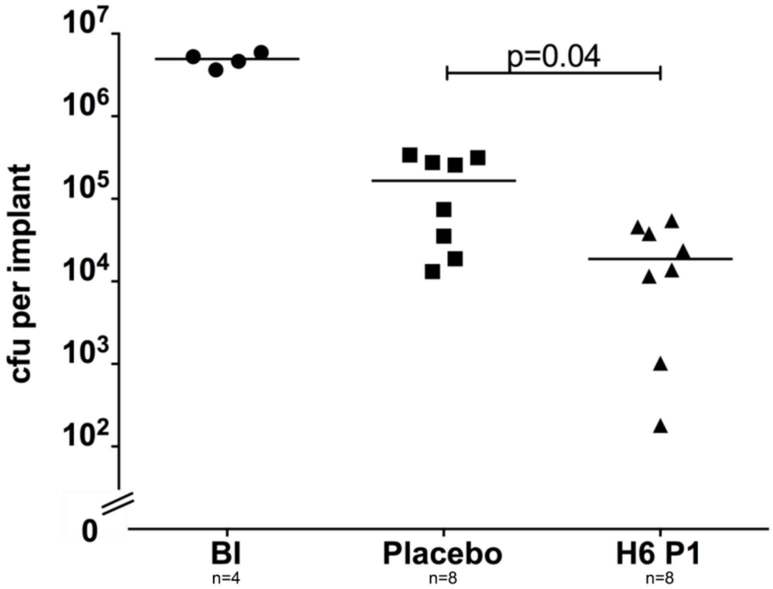

Supplementary Figure 4. The effect of treatment of *P. aeruginosa* biofilm infections with H6-335-P1. Silicone implants were incubated with *P. aeruginosa* for 20 hours for bacterial adhesion. At time zero, mice had the implants inserted in the peritoneal cavity. At 24 hours and 26 hours post-insertion of the implants (PI), the mice were treated with either H6-335-P1 (H6 P1) (6 μM corresponding to 1 μg compound per gram bodyweight) or vehicle (as placebo). At 28 hours PI the mice were euthanized and the CFU per implant was determined. Significance level is based on Mann-Whitney *U* test (analysis of non-parametric data). BI: Bacterial count before insertion of implants.

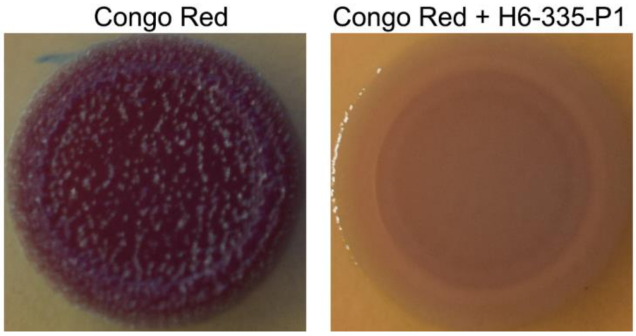

Supplementary Figure 5. Morphology of *P. aeruginosa wspF* macrocolonies on agar plates supplemented with Congo Red or Congo Red and H6-335-P1. Macrocolonies were grown from 5 μl overnight culture spotted on the agar plates. Photographs of the macrocolonies were acquired after 48 hours incubation at 30°C. Note that H6-335-P1 is yellow and colors the agar plates that are supplemented with this compound.

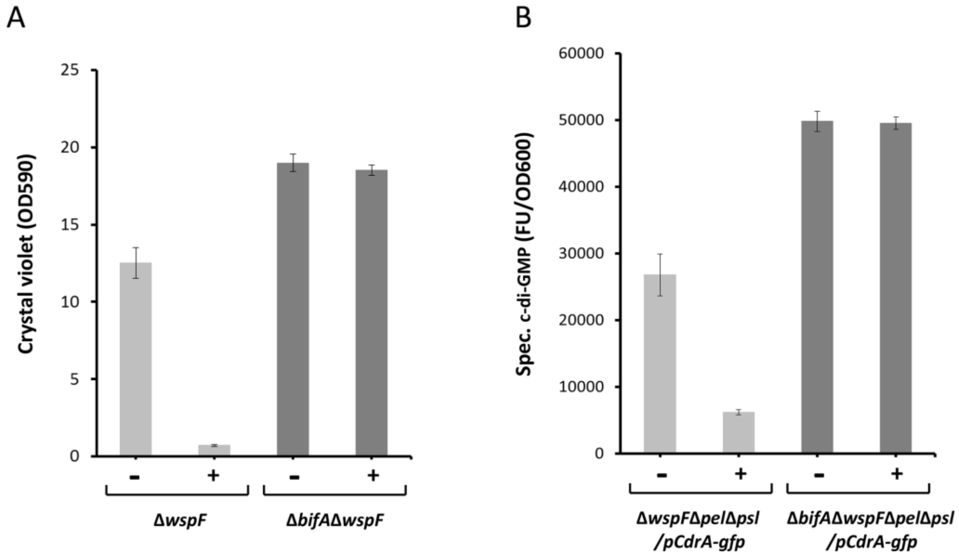

Supplementary Figure 6. (**A**) Amount of biofilm formed in microtiter trays by *P. aeruginosa ΔwspF* and *ΔbifAΔwspF* strains in the absence (-) or presence (+) of 100 μM H6-335-P1. Mean and standard deviation (bars) of three replicates are shown. (**B**) Fluorescence readout from *P. aeruginosa ΔwspFΔpelΔpsl*/pCdrA-gfp and *ΔbifAΔwspFΔpelΔpsl*/pCdrA-gfp cultures grown in the absence (-) or presence (+) of 100 μM H6-335-P1. Sampling was done after 17 hours of growth (early stationary phase). Mean and standard deviation (bars) of three replicates are shown.

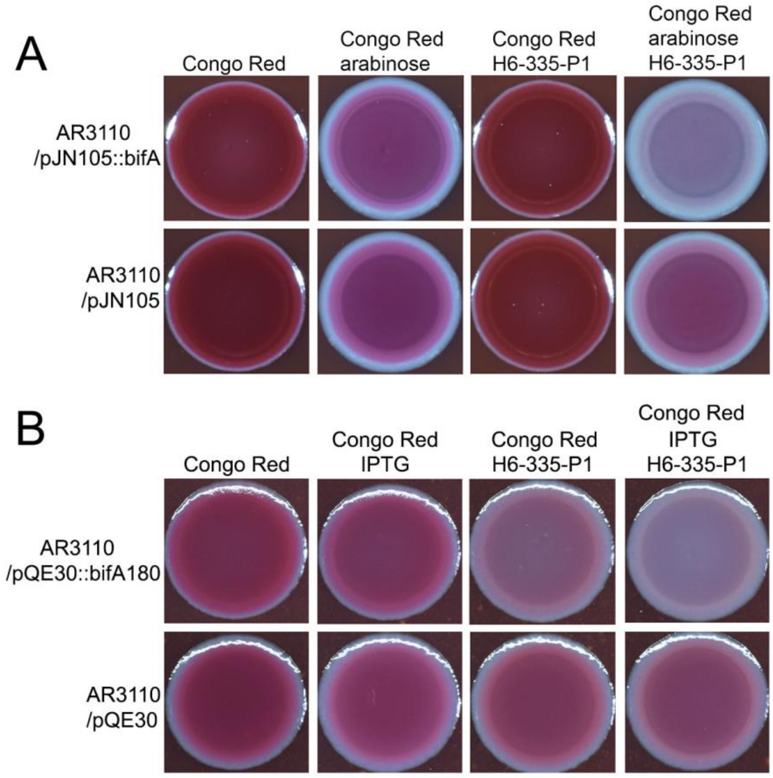

Supplementary Figure 7. Morphology of *E. coli* macrocolonies on agar plates. (**A**) Macrocolonies were grown from 5 μl overnight culture of *E. coli* AR3110/pJN105::bifA or *E. coli* AR3110/pJN105 (vector control) spotted on no-salt LB agar plates supplemented with respectively Congo Red (160 μg/ml), Congo Red and arabinose (0.2%), Congo Red and H6-335-P1 (100 μM), or Congo Red, arabinose and H6-335-P1. Photographs of the macrocolonies were acquired after 27 hours incubation at 28°C. (**B**) Macrocolonies were grown from 5 μl overnight culture of *E. coli* AR3110/pQE30::bifA180 or *E. coli* AR3110/pQE30 (vector control) spotted on agar plates supplemented with respectively Congo Red, Congo Red and IPTG (0.4 mM), Congo Red and H6-335-P1, or Congo Red, IPTG and H6-335-P1. Photographs of the macrocolonies were acquired after 24 hours incubation at 28°C.

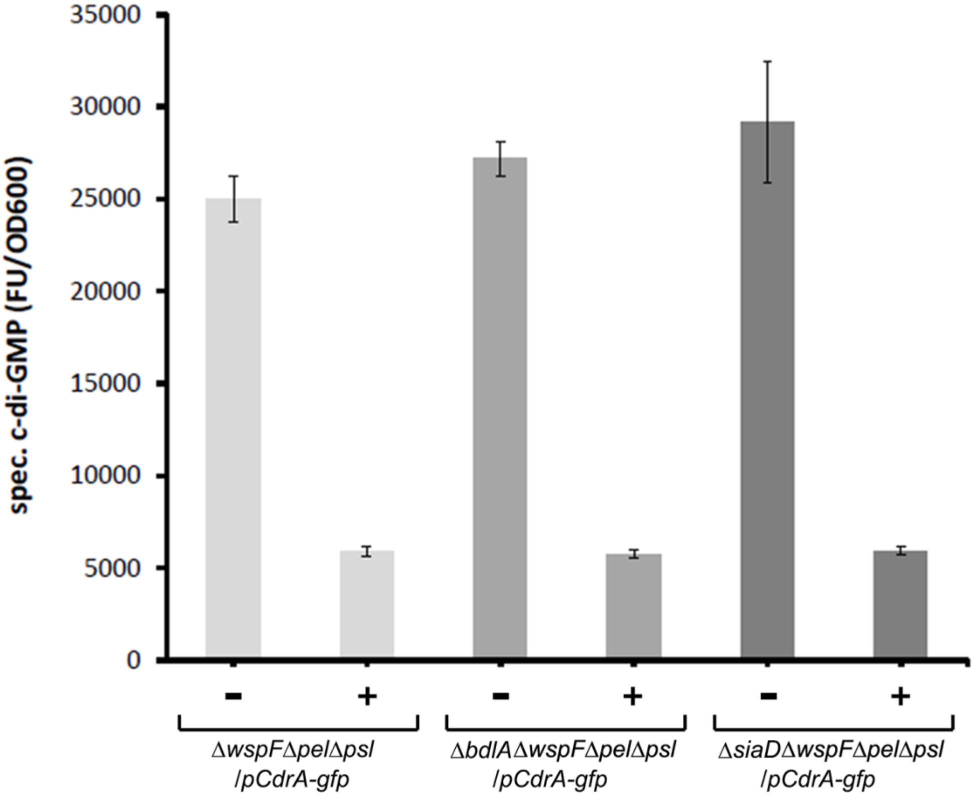

Supplementary Figure 8. Fluorescence readout from *P. aeruginosa ΔwspFΔpelΔpsl*/pCdrA-gfp, *ΔbdlAΔwspFΔpelΔpsl* /pCdrA-gfp and *ΔsiaDΔwspFΔpelΔpsl*/pCdrA-gfp cultures grown in the absence (-) or presence (+) of 100 μM H6-335-P1. Sampling was done after 17 hours of growth (early stationary phase). Mean and standard deviation (bars) of three replicates are shown.

Supplementary references

An S, Wu J, Zhang LH (2010). Modulation of *Pseudomonas aeruginosa* biofilm dispersal by a cyclic-di-GMP phosphodiesterase with a putative hypoxia-sensing domain. Appl Environ Microbiol **76**:8160-8173.

Andersen JB, Kragh KN, Hultqvist LD, Rybtke M, Nilsson M, Jacobsen TH, Givskov M., Tolker-Nielsen T (2021). Induction of native c-di-GMP phosphodiesterases leads to dispersal of *Pseudomonas aeruginosa* biofilms. Antimicrob Agents Chemother AAC.02431-20.

Chun K, Edenberg HJ, Kelley MR, Goebl MG (1997). Rapid amplification of uncharacterized transposon-tagged DNA sequences from genomic DNA. Yeast **13**:233-240.

Gjermansen M, Ragas P, Tolker-Nielsen T (2006). Proteins with GGDEF and EAL domains regulate *Pseudomonas putida* biofilm formation and dispersal. FEMS Microbiol Lett **265**:215-224.

Held K, Ramage E, Jacobs M, Gallagher L, Manoil C (2012). Sequence-Verified Two-Allele Transposon Mutant Library for *Pseudomonas aeruginosa* PAO1. J Bacteriol **194**:6387-6389.

Heydorn A, Nielsen AT, Hentzer M, Sternberg C, Givskov M, Ersbøll BK, Molin S (2000). Quantification of biofilm structures by the novel computer program COMSTAT. Microbiology **146**:2395-2407.

Hoang TT, Karkhoff-Schweizer RR, Kutchma AJ, Schweizer HP (1998). A broad-host-range Flp-FRT recombination system for site-specific excision of chromosomally-located DNA sequences: application for isolation of unmarked Pseudomonas aeruginosa mutants. Gene **212**:77-86.

Hoang TT, Kutchma AJ, Becher A, Schweizer HP (2000). Integration-proficient plasmids for Pseudomonas aeruginosa: site-specific integration and use for engineering of reporter and expression strains. Plasmid **43**:59-72.

Jakobsen TH, van Gennip M, Phipps RK, Shanmugham MS, Christensen LD, Alhede M, Skindersoe ME, Rasmussen TB, Friedrich K, Uthe F, Jensen PØ, Moser C, Nielsen KF, Eberl L, Larsen TO, Tanner D, Høiby N, Bjarnsholt T, Givskov M (2012). Ajoene, a sulfur-rich molecule from garlic, inhibits genes controlled by quorum sensing. Antimicrob Agents Chemother **56**:2314-2325.

Jacobs MA, Alwood A, Thaipisuttikul I, Spencer D, Haugen E, Ernst S, Will O, Kaul R, Raymond C, Levy R, Chun-Rong L, Guenthner D, Bovee D, Olson MV, Manoil C (2003). Comprehensive transposon mutant library of *Pseudomonas aeruginosa*. Proc Nat Acad Sciences **100**:14339-14344.

Kessler B, de Lorenzo V, Timmis KN (1992). A general system to integrate lacZ fusions into the chromosomes of gram-negative eubacteria: regula- tion of the Pm promoter of the TOL plasmid studied with all controlling elements in monocopy. Mol Gen Genet **233**:293-301.

Köhler T, van Delden C, Curty LK, Hamzehpour MM, Pechere JC (2001). Overexpression of the MexEFOprN multidrug efflux system affects cell-to-cell signaling in *Pseudomonas aeruginosa*. J Bacteriol **183**:5213-5222.

Kovach ME, Elzer PH, Hill DS, Robertson GT, Farris MA, Roop RM, Peterson KM (1995). Four new derivatives of the broad-host-range cloning vector pBBR1MCS, carrying different antibiotic resistance cassettes. Gene **166**:175-176.

Kulasekara HD (2014). Transposon mutagenesis. Methods Mol Biol **1149**:501-519.

Newman JR, Fuqua C (1999). Broad-host-range expression vectors that carry the L-arabinose-inducible *Escherichia coli araBAD* promoter and the araC regulator. Gene **227**:197-203.

Rybtke M, Berthelsen J, Yang L, Høiby N, Givskov M, Tolker-Nielsen T (2015). The LapG protein plays a role in *Pseudomonas aeruginosa* biofilm formation by controlling the presence of the CdrA adhesin on the cell surface. MicrobiologyOpen. **4**:917-930.

Rybtke MT, Borlee BR, Murakami K, Irie Y, Hentzer M, Nielsen TE, Givskov M, Parsek MR, Tolker-Nielsen T (2012). Fluorescence-based reporter for gauging cyclic di-GMP levels in *Pseudomonas aeruginosa*. Appl Environ Microbiol **78**:5060-5069.

Serra DO, Richter AM, Hengge R (2013). Cellulose as an architectural element in spatially structured *Escherichia coli* biofilms. J Bacteriol. **195**:5540-54.

Simon R, Priefer U, Puhler A (1983). A broad host range mobilization system for in vivo genetic engineering transposon mutagenesis in gram-negative bacteria. BioTechnol **1**:784-791.

Stover CK, Pham XQ, Erwin AL, Mizoguchi SD, Warrener P, Hickey MJ, Brinkman F S, Hufnagle WO, Kowalik DJ et al. (2000). Complete genome sequence of *Pseudomonas aeruginosa* PAO1, an opportunistic pathogen. Nature **406**:959-964.
